# Supplementary material for: Computational model of flower pattern evolution predicts spontaneous emergence of boundary cell types across the petal epidermis
Source: Development. 2026 Jun 22;153(12):dev205745. doi: 10.1242/dev.205745 (PMC13354956; doi:10.1242/dev.205745)
Supplement: Supplementary information [file develop-153-205745-s1.pdf]

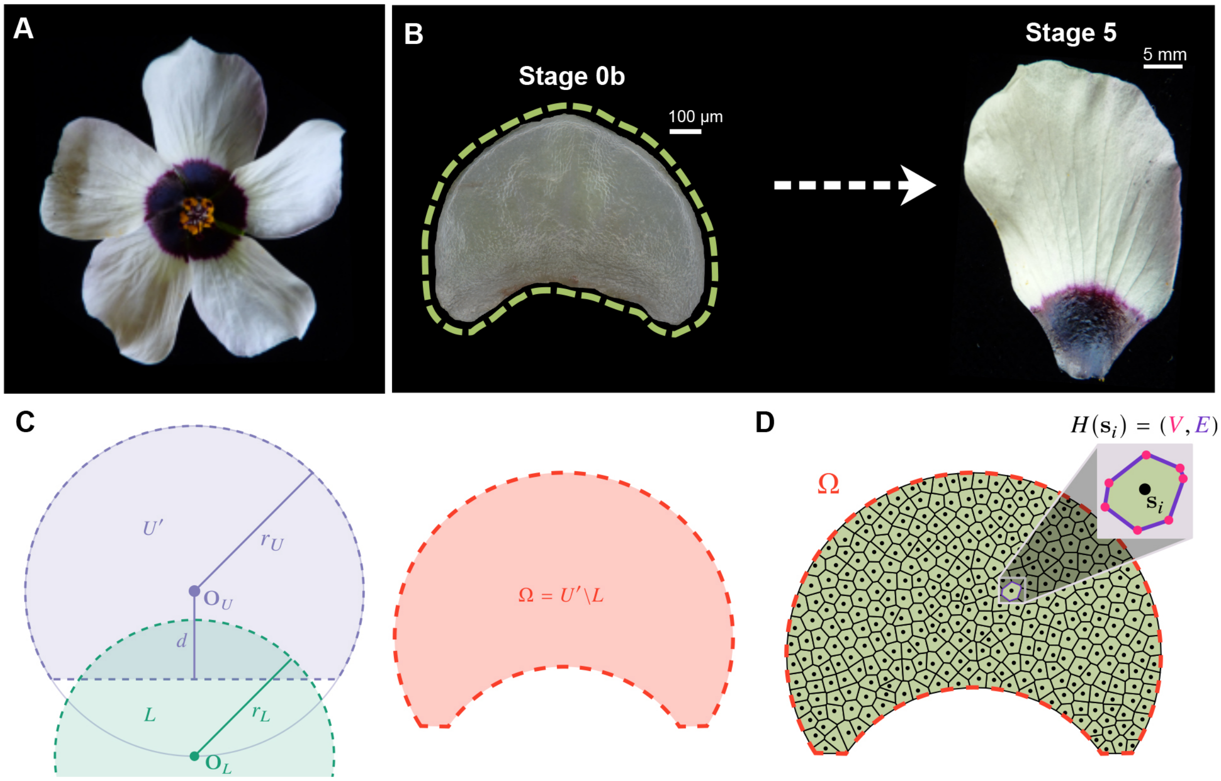

Fig. S1. Approximation of the *H. trionum* petal adaxial epidermis tissue shape at developmental Stage 0b. (**A**) The flower of *H. trionum* features a bullseye pattern on its corolla. (**B**) Cell differentiation across the adaxial epidermis of the *H. trionum* petal primordium leads to bullseye pattern formation during petal development. As the petal is likely pre-patterned at an early developmental stage, we created a cell-based developmental model of the Stage 0b petal. (**C**) The Stage 0b tissue shape is approximated by the difference between the major segment of a larger upper circle U' and a smaller lower circle L.(**D**) The resulting petal tissue domain Ω is discretised into $N_C = 320$ cells through Voronoi tessellation. Each Voronoi cell and its Voronoi edges are drawn along with a dot representing its site (see Methods).

Development • Supplementary information

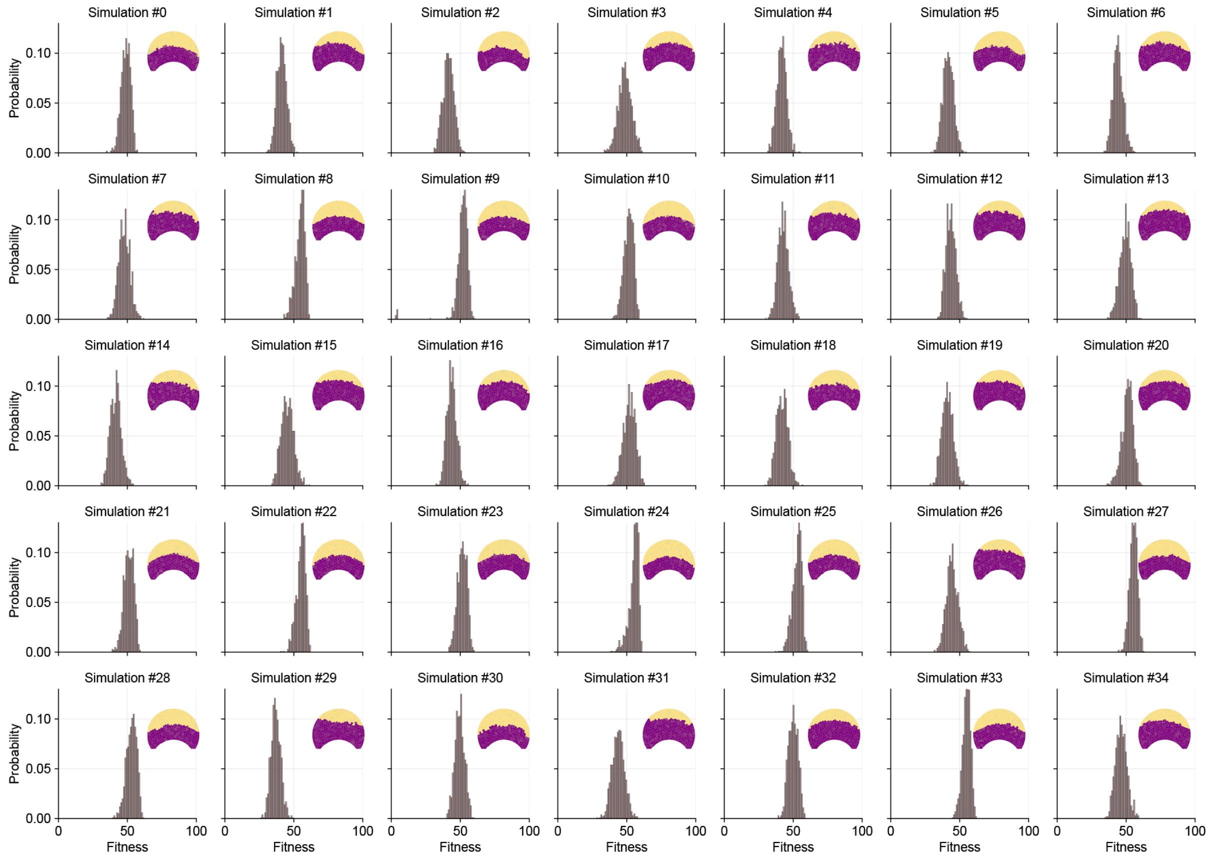

Fig. S2. All 35 populations evolved gene regulatory networks (GRNs) that robustly generate bullseye patterns. For each simulation, we tested the developmental robustness of the fittest evolved individual by repeating its development 1000 times, each time using a different tissue morphology (see Methods).

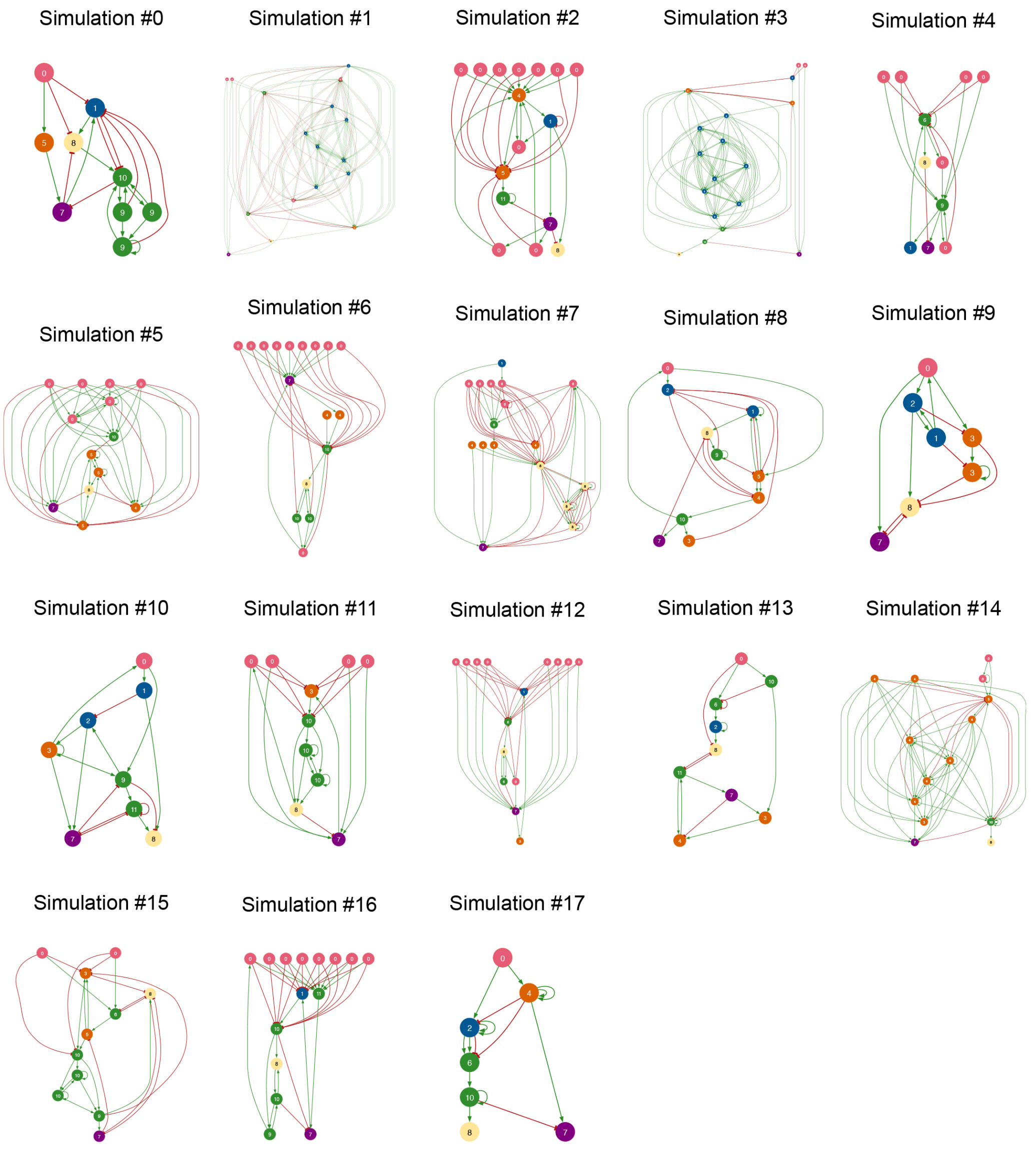

Fig. S3. Pruned GRN of the fittest individual in the final generation for simulations 0 to 17.

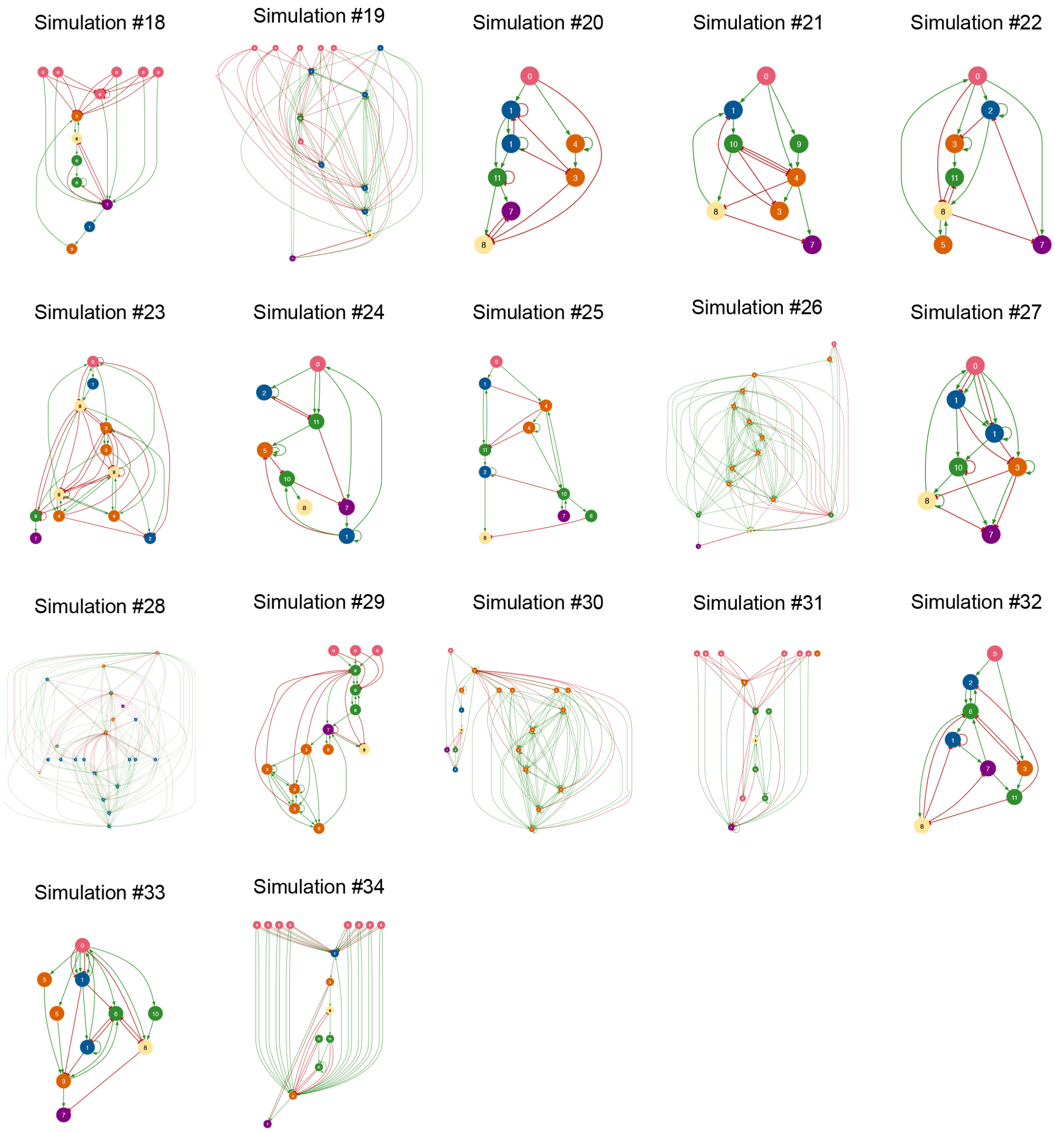

Fig. S4. Pruned GRN of the fittest individual in the final generation for simulations 18 to 34.

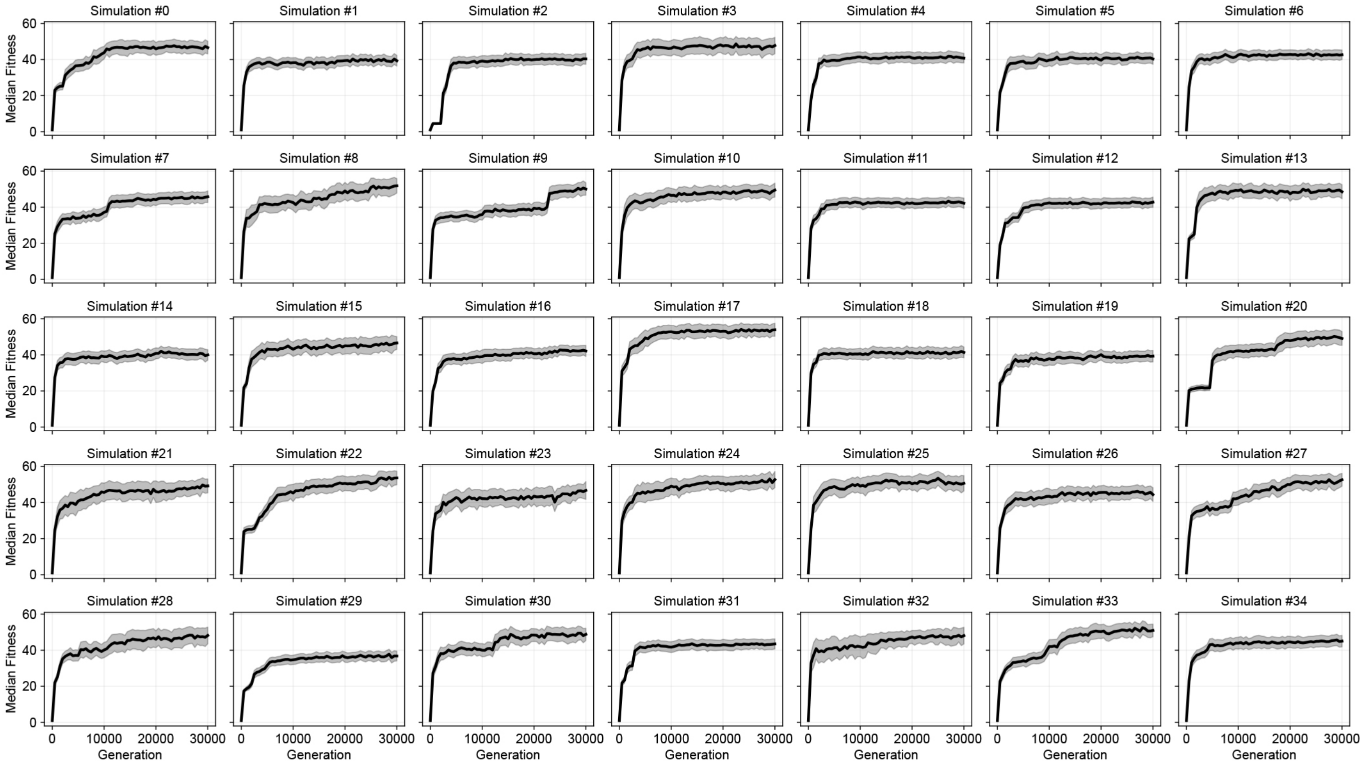

Fig. S5. Evolutionary dynamics of population median fitness (solid black line) for all 35 simulations. The median fitness of the population consisting of 1000 individuals along with the interquartile range (IQR, grey shaded region) is shown for each simulation.

Development • Supplementary information

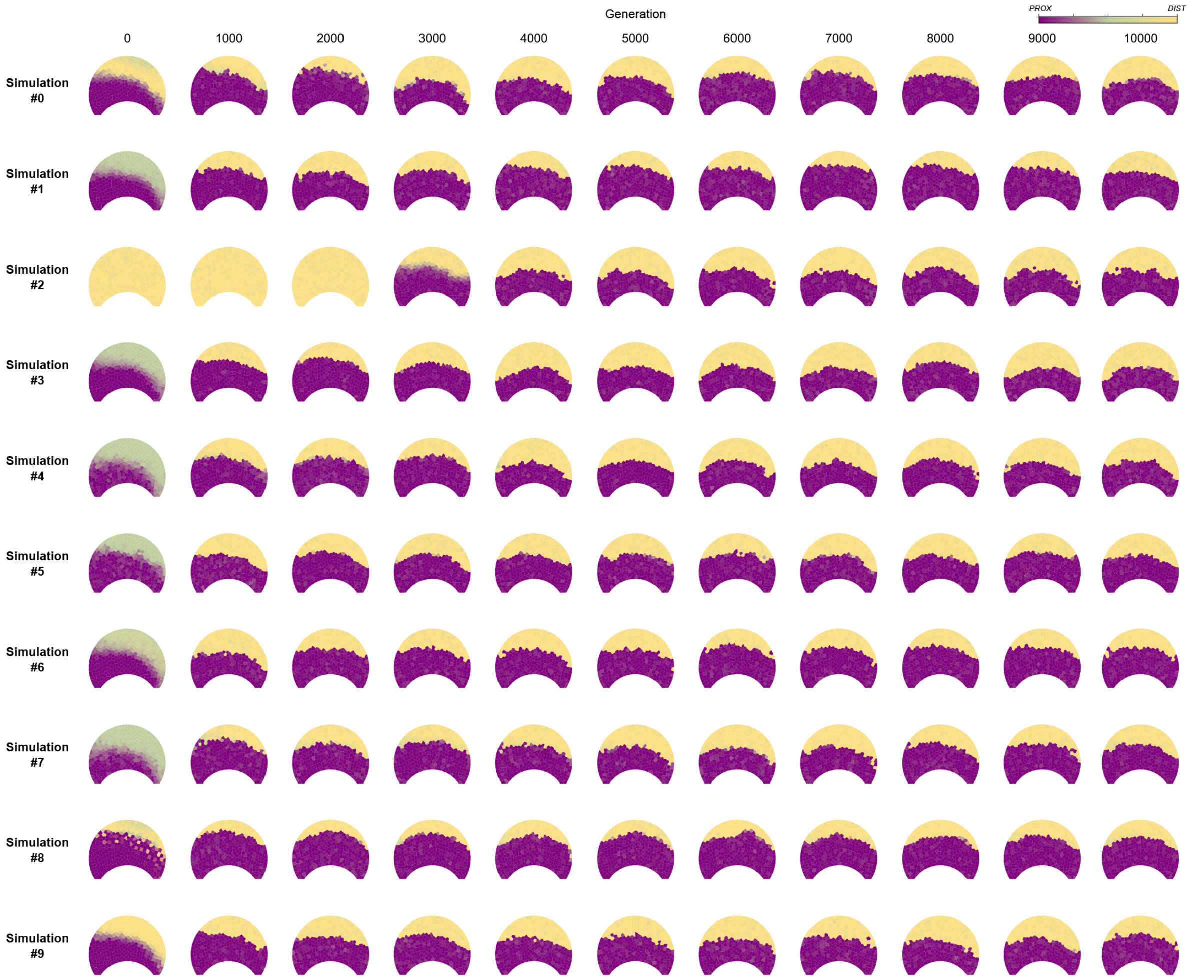

Fig. S6. Phenotype evolution of early generations (generations 0 until 10000) of 10 simulations.

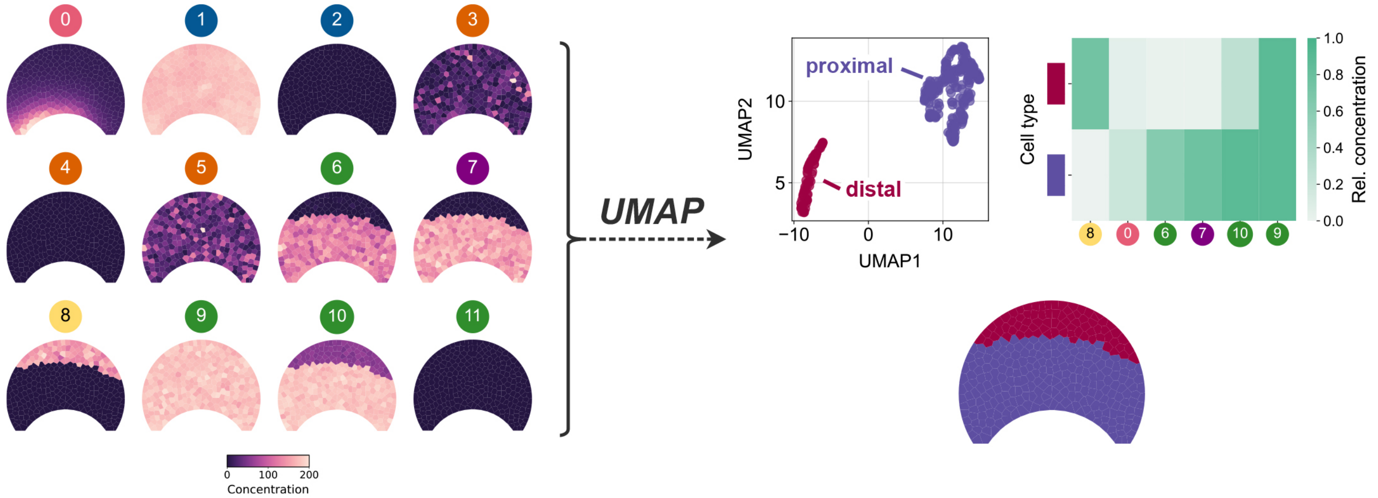

Fig. S7. UMAP dimensionality reduction followed by HDBSCAN clustering reveals cell types across petal. UMAP dimensionality reduction was applied to normalized protein concentrations after filtering out low-relevance proteins. HDBSCAN clustering was then performed to identify cell types, which are visualized in a heatmap and across the petal's epidermal cells. Refer to Methods: Cell Types and Dimensionality Reduction and Clustering for more information on this analysis.

Development • Supplementary information

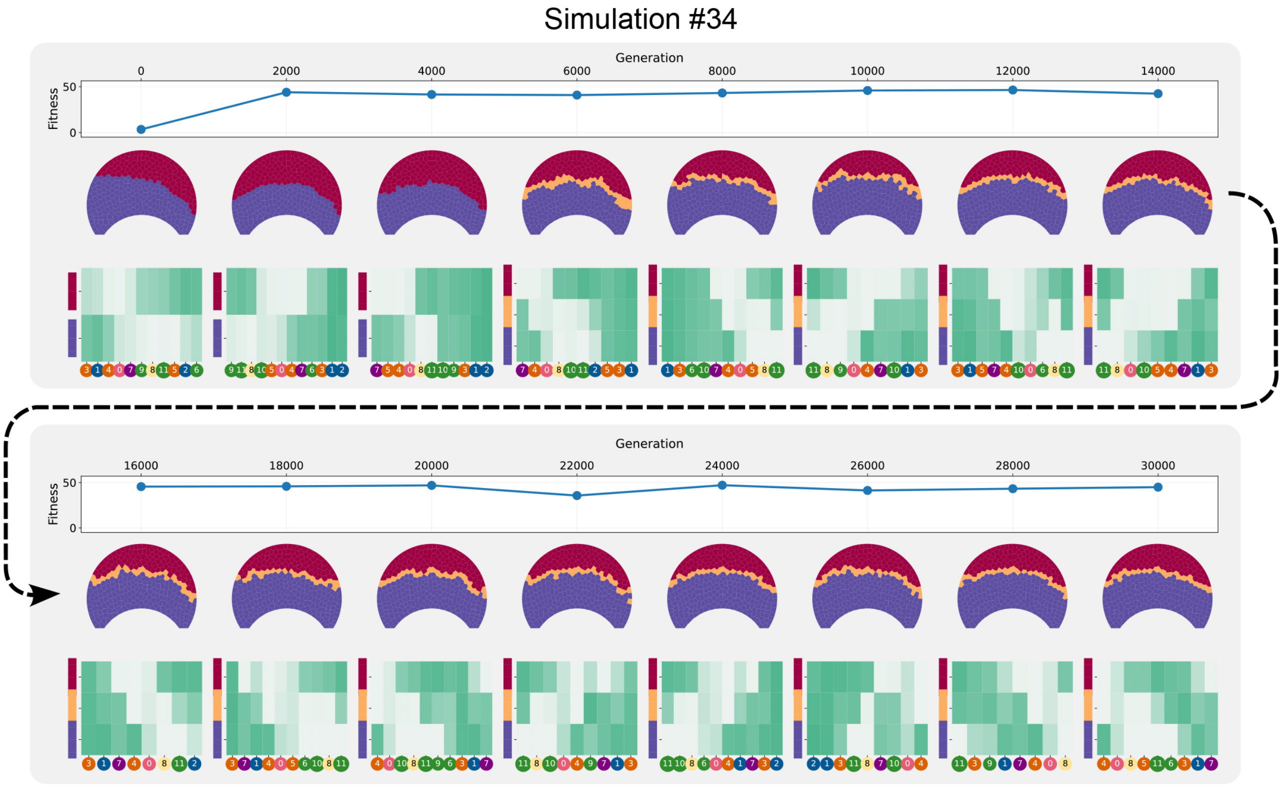

Fig. S8. Evolutionary trajectory of the fittest individual in the final population of Simulation #34. Shown are the ancestors of the individual with highest fitness in generation 30000, together with their fitness and cell types.

Development • Supplementary information

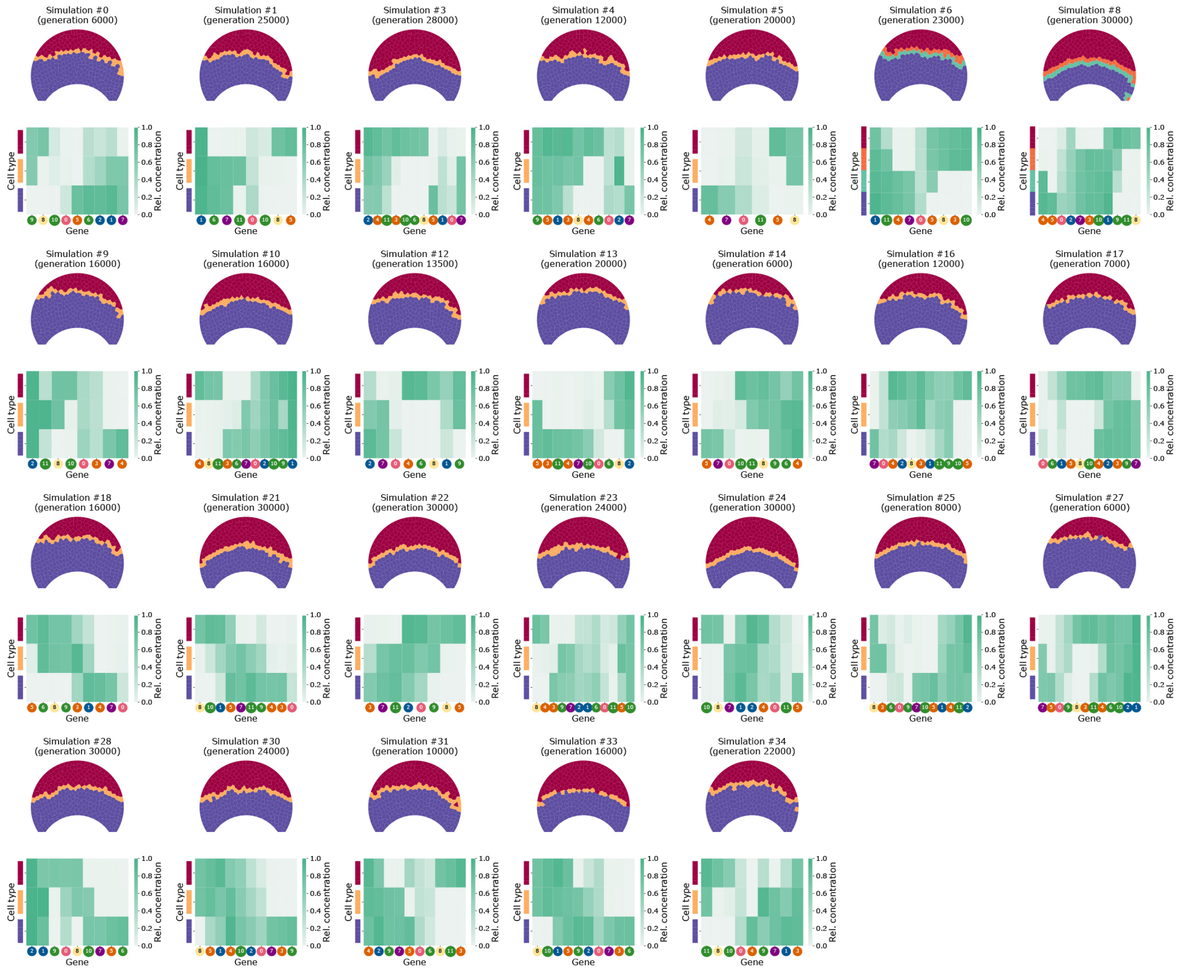

Fig. S9. Overview of all noisy simulations that evolved boundary cell types. For each simulation, a single sample from one generation along the ancestral lineage is shown. Bullseye boundary cell types typically appeared across multiple generations in most simulations (Fig. ??).

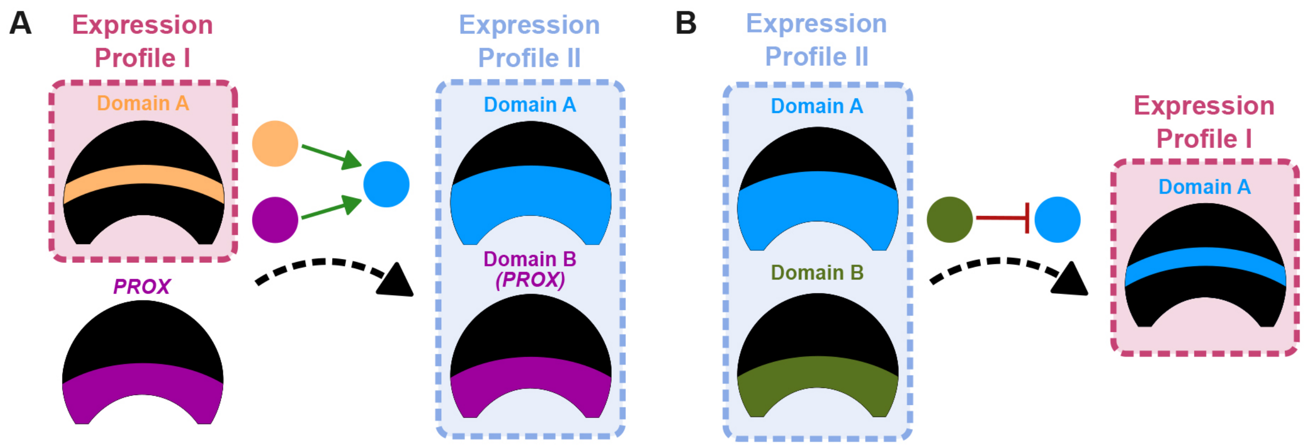

Fig. S10. Each boundary expression profile may lead to the emergence of the other. (**A**) Expression profile I can lead to expression profile II by activation of a gene by both the *PROXIMAL IDENTITY GENE (PROX)* and boundary gene.(**B**) Expression profile II can lead to expression profile I when the gene expressed in the smaller bullseye domain (Domain B) inhibits the gene expressed in the larger bullseye domain (Domain A).

Development • Supplementary information

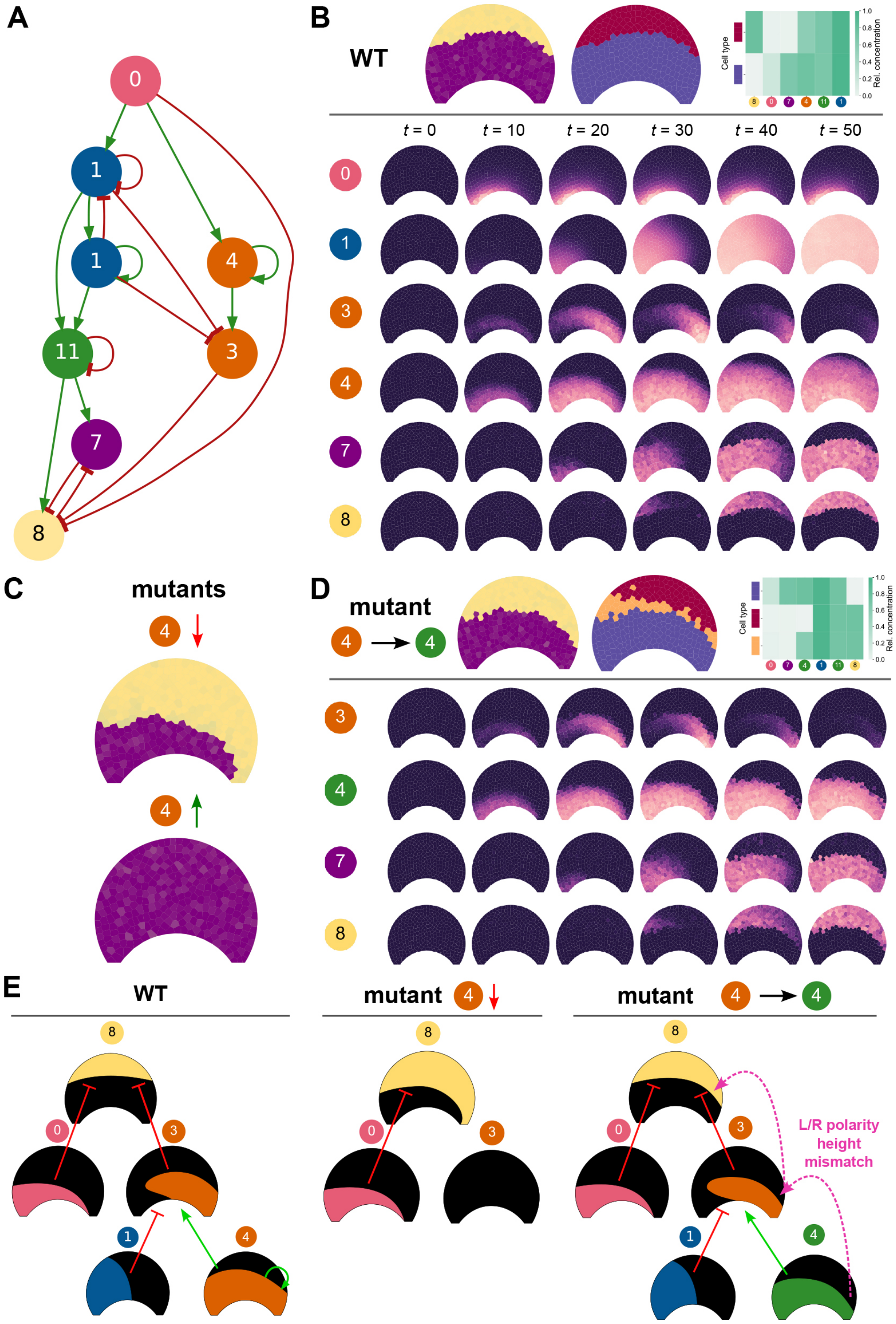

Fig. S11. Mechanism of symmetric bullseye patterning without boundary cell type.

(**A**) Pruned GRN of an individual from a representative simulation with no boundary cell type (Simulation #20) at generation 30000. (**B**) Wild-type phenotype and early patterning dynamics. In this GRN, genes 1 and 4 cooperate to establish a temporal right-polarity expression pattern in gene 3, balancing the left-polarity signal in gene 0. Genes 0 and 3 then integrate into gene 8 to produce a symmetric distal bullseye pattern. (**C**) Phenotypic effects of gene 4 knockout and overexpression, resulting in bullseye asymmetry and bullseye loss, respectively. (**D**) Mutant in which gene 4 is converted from a cell-cell communication gene to a transcription factor (TF), with early patterning dynamics shown for downstream genes. (**E**) Cartoon summarising how bilateral symmetry is established in this GRN. In wild type (WT), genes 1 and 4 establish right-polarity expression in gene 3, complementing the left-polarity signal in gene 0 to produce symmetric distal bullseye expression in gene 8. Knockout of gene 4 prevents gene 3 activation, leaving the right side of the bullseye unfilled. When gene 4 is converted to a TF, its self-activation range is reduced: gene 3 is still expressed but the length of its expression domain along the proximo-distal petal axis no longer matches that of gene 0, resulting in a slightly asymmetric bullseye.

Development • Supplementary information

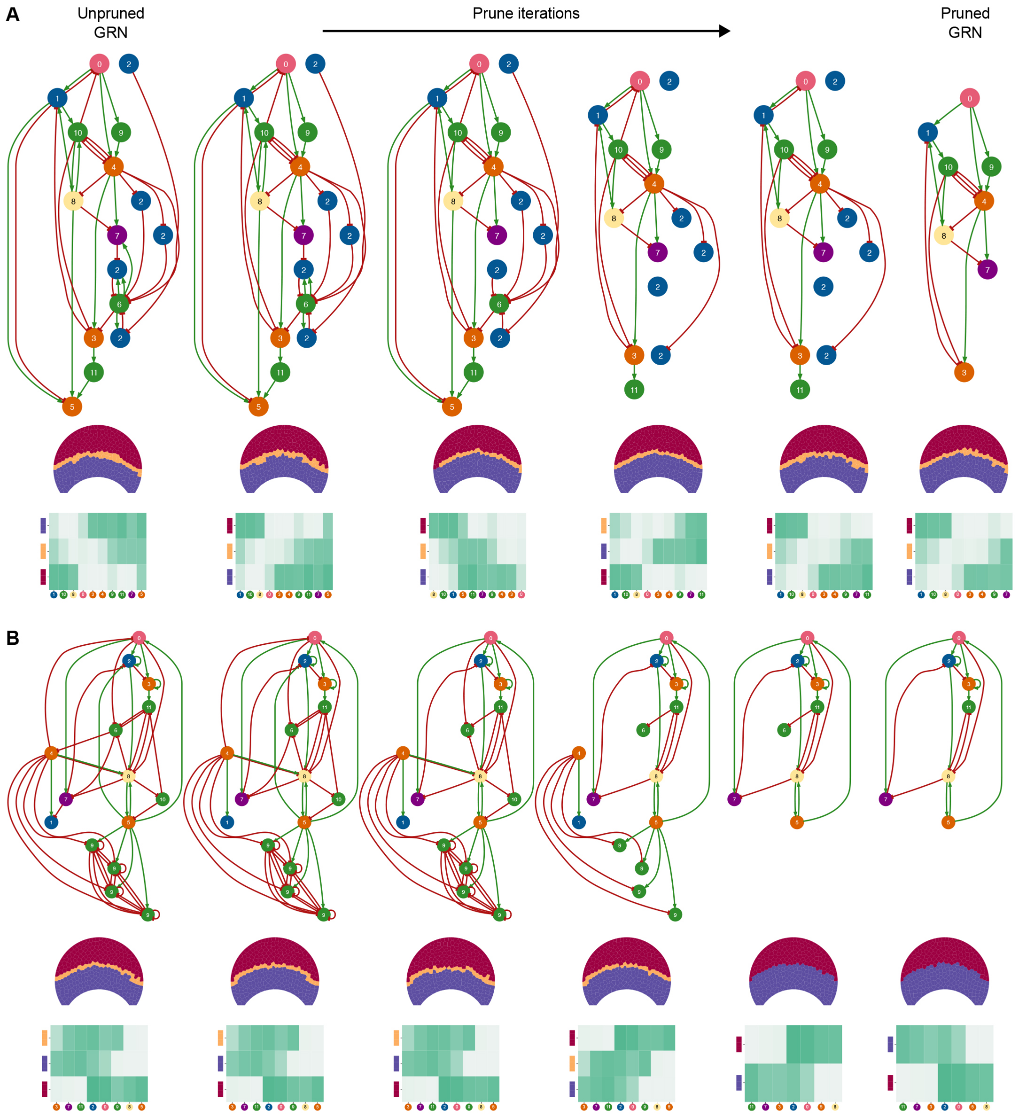

Fig. S12. Depiction of the pruning process on two evolved GRNs. When pruning a GRN, we iteratively delete a single gene or interaction and redevelop the pattern 20 times. If the average fitness remains within 3% of the original average fitness score, the deletion is accepted and a next deletion is attempted within the reduced GRN. (**A**) Pruning on a GRN whose boundary cell type persists after pruning. (**B**) Pruning on a GRN whose boundary cell type gets lost after pruning. After pruning, GRNs typically only use a subset of the total 12 gene types available.

Development • Supplementary information

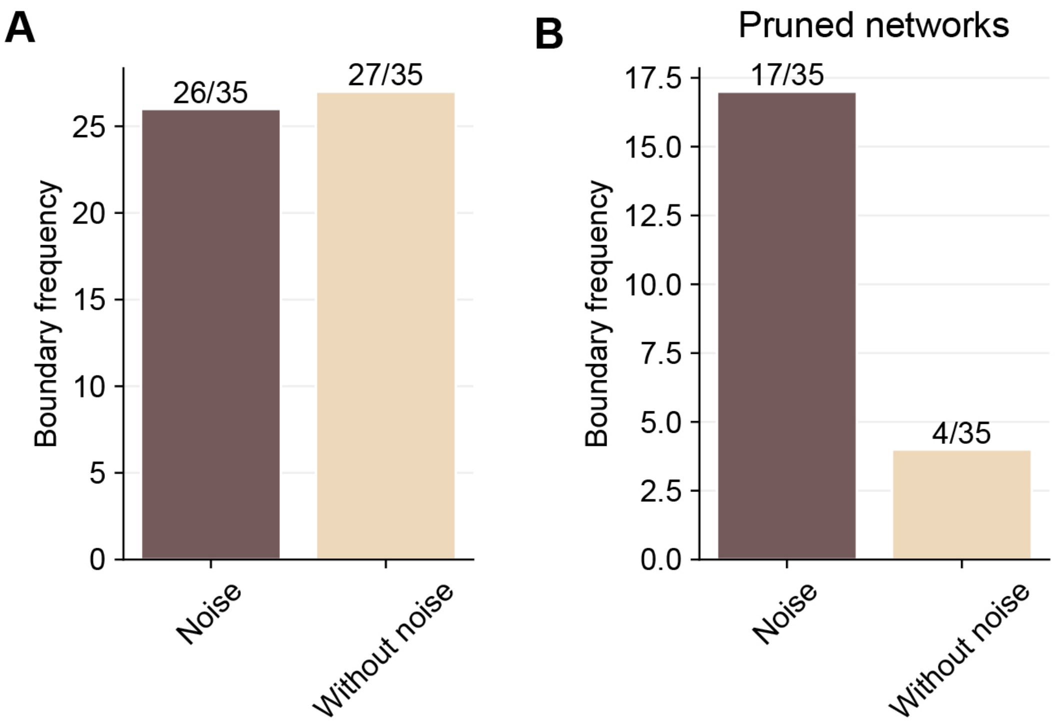

Fig. S13. Frequency of evolved boundary cell types across lineages before and after pruning GRNs.

(**A**) Number of simulations that evolved a boundary cell type in their lineage, comparing simulations with and without noise. (**B**) Number of networks that have a boundary cell type after pruning (see Methods), comparing simulations with and without noise.

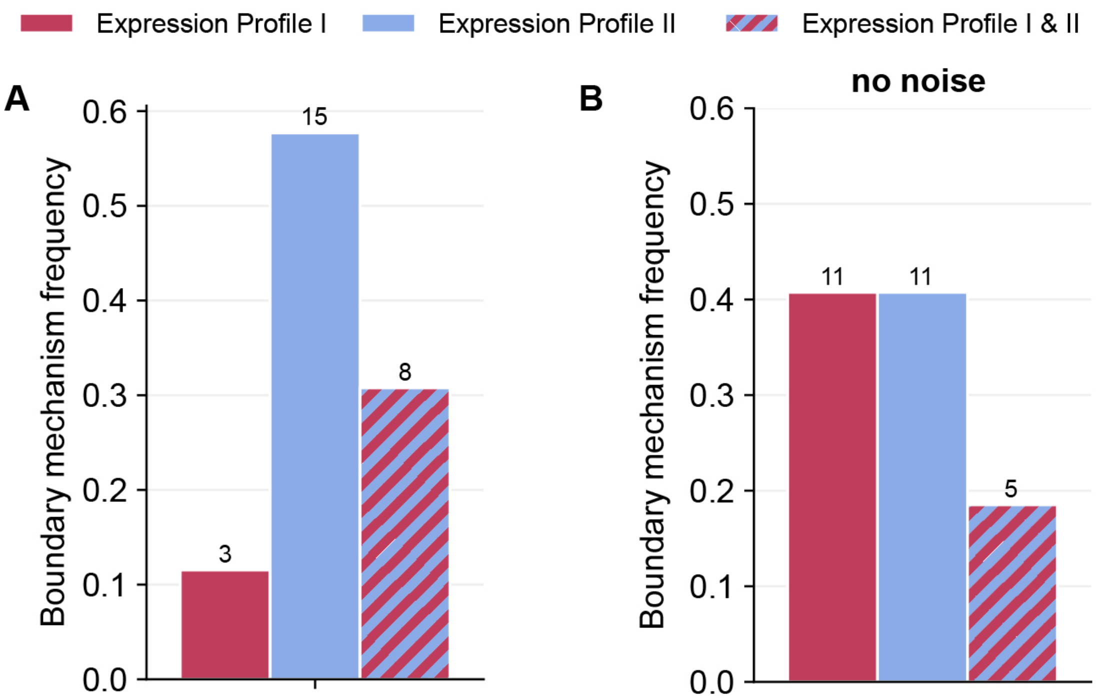

Fig. S14. Distribution of boundary expression profiles in the simulations where a boundary cell type appeared in the model with molecular noise (A) and without (B). The absolute number of occurrences for each expression profile is indicated above each bar.

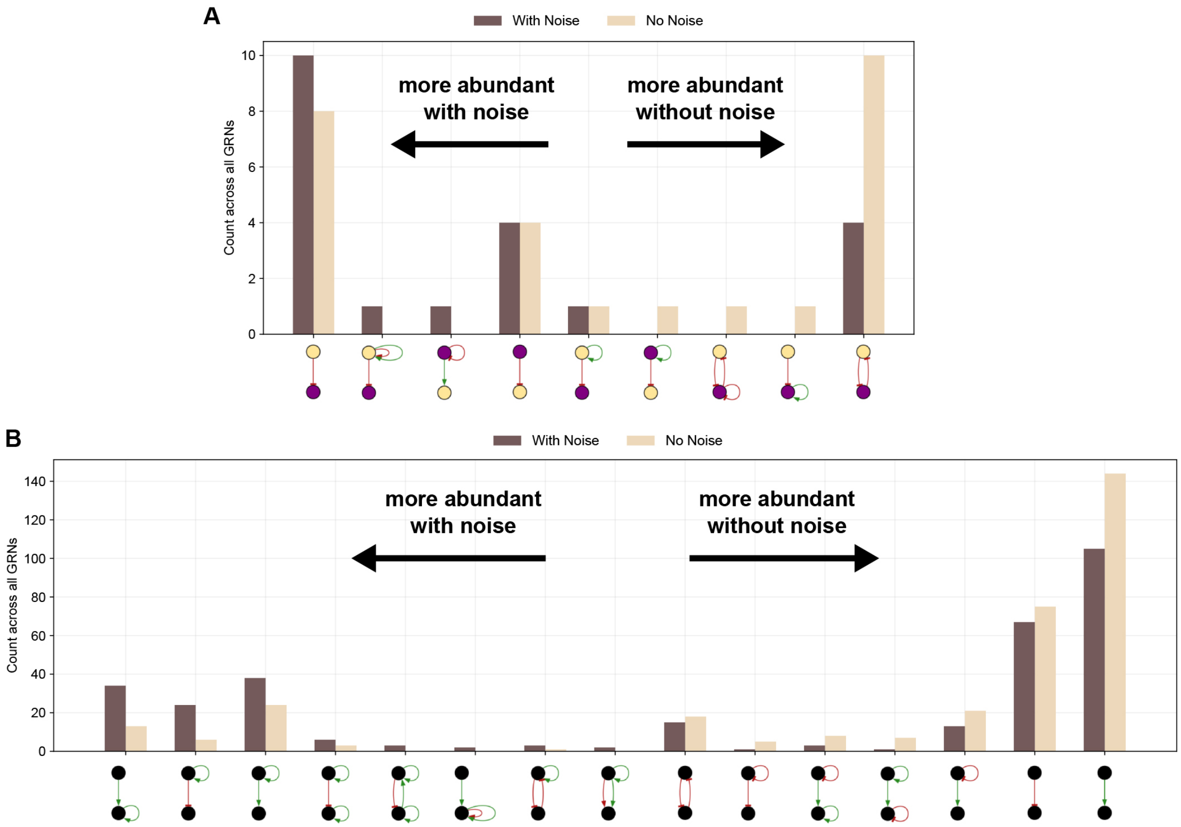

Fig. S15. Differences in GRN motifs between simulations with and without noise. (**A**) Differences between 2-motifs containing *DIST* (cream) and *PROX* (purple) genes. (**B**) Differences between 2-motifs of any combination of genes. Note that in simulations with noise, certain motifs are overrepresented due to extensive gene duplications (see Figs S3 and S4).

Development • Supplementary information

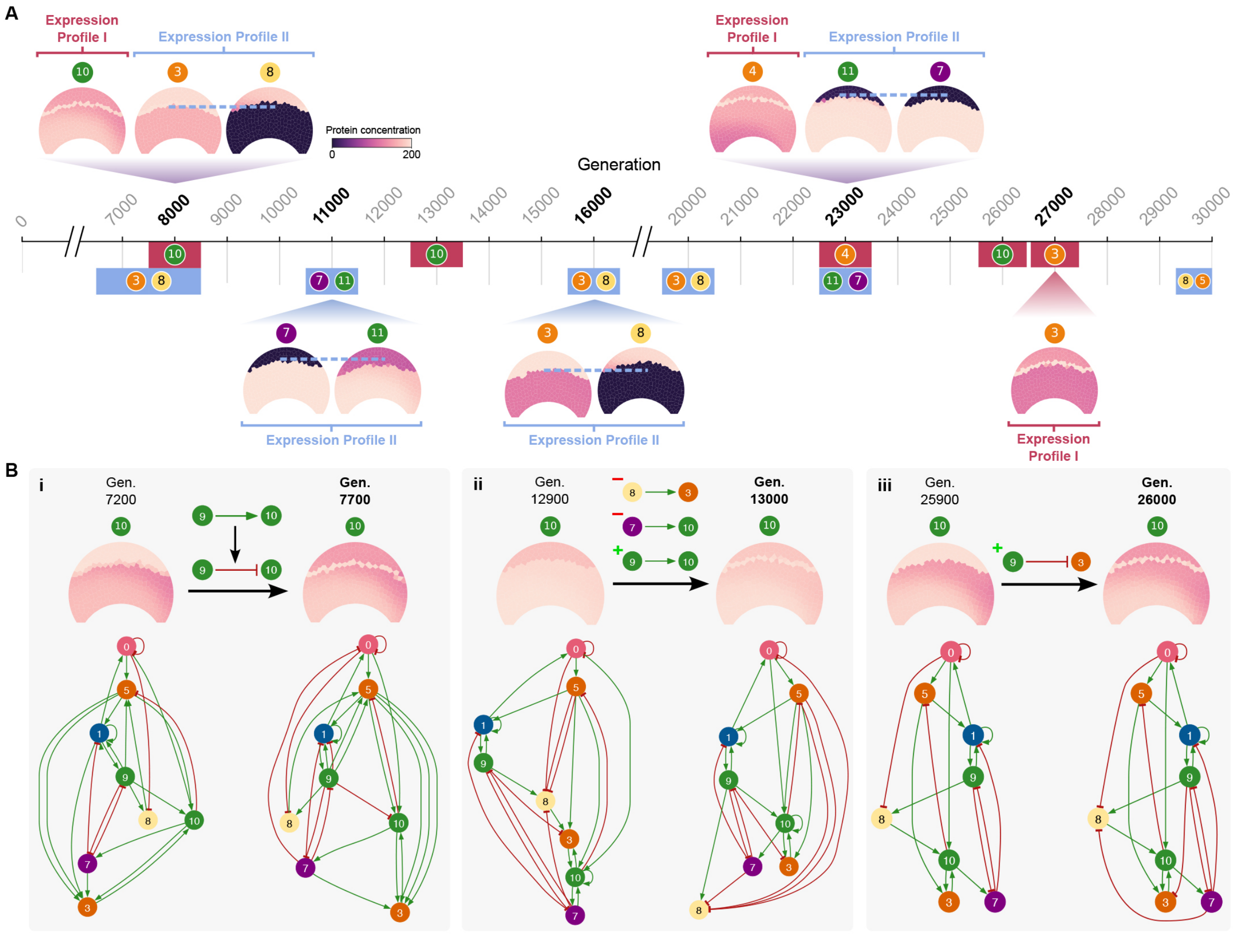

Fig. S16. Highly transient evolution of boundary cell type.

(**A**) Evolution of boundary cell types in deterministic Simulation #13, illustrating the diversity of boundary mechanisms that evolved within a single simulation. The presence of boundary expression profile I is depicted in pink, while boundary expression profile II is shown in blue. The gene(s) responsible for each emergent boundary cell type are displayed alongside the respective mechanism: for expression profile I, the gene preferentially expressed in the boundary; for expression profile II, the two genes with overlapping expression domains of uneven lengths. Expression patterns of these genes are shown at selected generations. (**B**) For each independent emergence of the boundary cell type mediated by gene 10 (expression profile I), we identify the final mutation(s) responsible. The first appearance at generation 7700 (i) results from a single transcription factor binding site (TFBS) weight inversion; the second at generation 13000 (ii) from three mutations (two TFBS deletions and one TFBS insertion); and the third at generation 27000 (iii) from a single TFBS insertion. All GRNs shown are unpruned networks with leaf and unreached nodes removed (i.e., genes that do not regulate other genes or are never activated) for visual clarity.

Development • Supplementary information

Table S1. Static parameters for the evolutionary developmental model. Units: M, arbitrary molecular unit; h, developmental time unit (hour); s, second. Note: $D_{j,m}$ [µm^2 s^{-1}] is computed as $D_{j,m} = D_0 \cdot w_{j,m}$, where $w_{j,m}$ [µm] is the contact wall length between adjacent cells (Eqn **??**), and D_0 [µm s^{-1}] is the base diffusion rate, which incorporates the assumption of constant wall thickness. The range of values here are based on the minimum and maximum contact wall lengths in the tissue meshes.

Parameter	Description	Value
**Developmental**		
N_C	Number of cells in tissue	320
N_{morph}	Number of distinct petal morphologies	1000
D_0	Protein diffusion base rate	0.0148 µm s^{-1}
$D_{j,m}$	Effective protein diffusion coefficient	$[0.16, 0.62]$ µm^2 s^{-1}
γ	Protein decay rate	0.2 h^{-1}
S_0	Baseline constitutive signal expression	600 Mh^{-1}
T_D	Integration time	140 h
Δt	Integration step size	0.2 h
n_{end}	Number of discrete integration steps ($T_D/\Delta t$)	700
n_{start}	Discrete time step at which to start sampling fitness	500
**Evolutionary**		
N_{pop}	Population size	1000
T_{gen}	Number of generations	30000
α_{init}	Initial maximum transcription rate of a gene	25 Mh^{-1}
λ_{init}	Initial decay rate of gene mRNA	0.2 h^{-1}
K_{init}	Initial dissociation constant of a TFBS	40 M
σ_α	Std. dev. for maximum transcription rate mutations	5 Mh^{-1}
σ_λ	Std. dev. for mRNA decay rate mutations	0.01 h^{-1}
σ_K	Std. dev. for dissociation constant mutations	10 M
P_{genedup}	Probability of gene duplication	0.004 per gene
P_{genedel}	Probability of gene deletion	0.006 per gene
$P_{\text{genetransc}}$	Probability of changing a gene's maximum transcription rate	0.001 per gene
$P_{\text{genedecay}}$	Probability of changing a gene's mRNA decay rate	0.001 per gene
P_{TFBSdup}	Probability of TFBS duplication	0.0011 per TFBS
P_{TFBSdel}	Probability of TFBS deletion	0.0015 per TFBS
$P_{\text{TFBSchange}}$	Probability of changing the dissociation constant of a TFBS	0.004 per TFBS
P_{TFBStype}	Probability of changing the gene type of a TFBS	0.0004 per TFBS
P_{TFBSinv}	Probability of inverting the weight of a TFBS	0.0004 per TFBS
P_{TFBSnew}	Probability of inserting a new random TFBS	0.032

Table S2. Evolvable parameters and their allowed ranges for the developmental model.

Parameter	Description	Value range
α_i	Maximum transcription rate of gene i	$[5, 80]$ Mh^{-1}
λ_i	mRNA decay rate of gene i	$[0.05, 0.9]$ h^{-1}
$K_{k,i}$	Dissociation constant of TFBS k of gene i	$[5, 100]$ M
$W_{k,i}$	Regulatory effect (inhibiting or activating) of gene k on gene i	$\{-1, 1\}$

Table S3. Differential gene expression analysis between proximal and boundary regions of Stage 2 *H. trionum* petal primordia.

Available for download at
https://journals.biologists.com/dev/article-lookup/doi/10.1242/dev.205745#supplementary-data

Table S4. Differential gene expression analysis between distal and boundary regions of Stage 2 *H. trionum* petal primordia.

Available for download at
https://journals.biologists.com/dev/article-lookup/doi/10.1242/dev.205745#supplementary-data

Table S5. Genes preferentially expressed in the petal bullseye boundary region at Stage 2. Log2 fold Change < -1 or > 1 and $p_{\text{adj}} < 0.05$.

Available for download at
https://journals.biologists.com/dev/article-lookup/doi/10.1242/dev.205745#supplementary-data

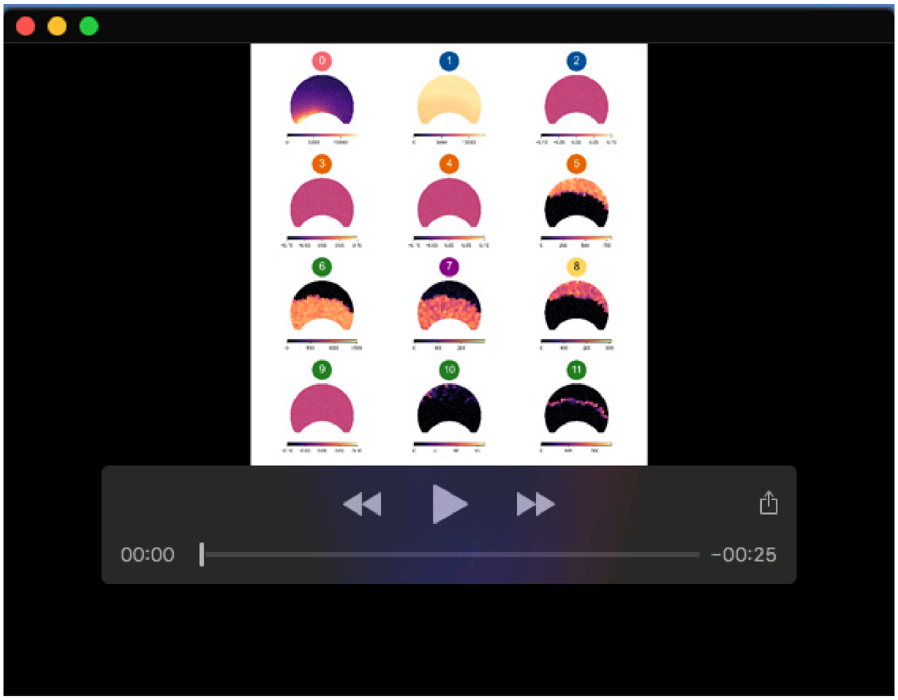

Movie 1. Representative developmental mechanism which creates a boundary cell type by expression profile I. Gene 11 is preferentially expressed in the boundary region, leading to a boundary cell type through expression profile I.

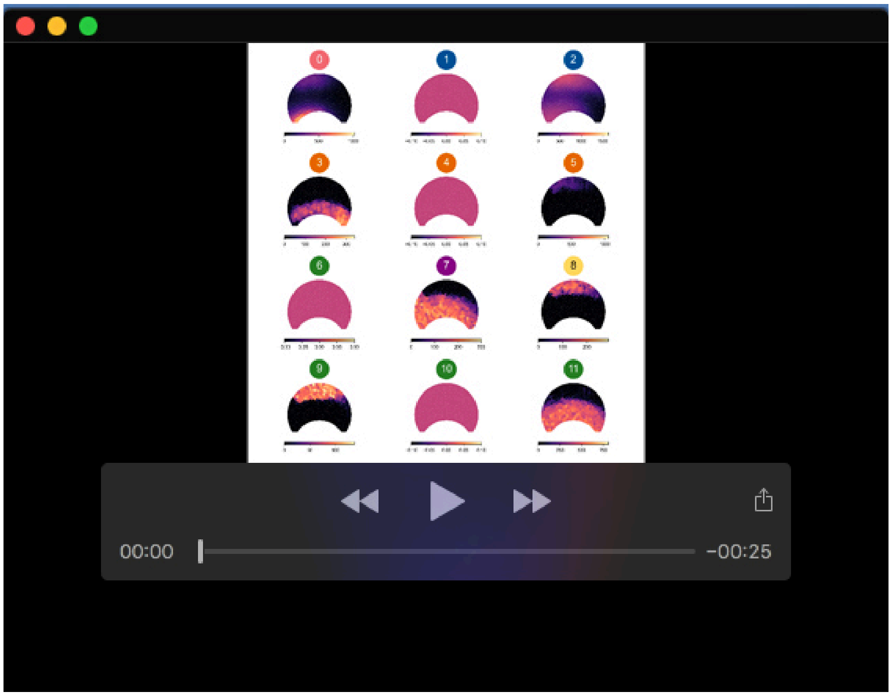

Movie 2. Representative developmental mechanism which creates a boundary cell type by expression profile II. Gene 3 and 7 are expressed in proximal domains of different sizes, leading to a boundary cell type through expression profile II.

Development • Supplementary information
