# Supplementary material for: Computational model of flower pattern evolution predicts spontaneous emergence of boundary cell types across the petal epidermis
Source: Development. 2026 Jun 22;153(12):dev205745. doi: 10.1242/dev.205745 (PMC13354956; doi:10.1242/dev.205745)
Supplement: Supplementary file 1 [file develop-153-205745_review_history.pdf]

Computational model of flower pattern evolution predicts spontaneous emergence of boundary cell types across the petal epidermis

Steven Oud, Maciej M. Żurowski, Pjotr L. van der Jagt, May T. S. Yeo, Joseph F. Walker, Edwige Moyroud and Renske M.A. Vroomans

DOI: 10.1242/dev.205745

Editor: Dominique Bergmann
Review timeline

Submission to Review Commons:	28 November 2025
Submission to Development:	17 April 2026
Editorial Decision:	12 May 2026
First revision received:	15 May 2026
Accepted:	18 May 2026

Reviewer 1:

Evidence, reproducibility and clarity

The manuscript presents the findings of a computational investigation, whereby populations of artificial "genomes" and their products are evolved algorithmically. They are subjected to a fitness constraint defined in terms of a spatial expression pattern on a petal shaped template. The specific focus of this work is the formation of two-pigment patterns on flower petals, which give rise to "bullseye" patterned flowers. A computational survey suggests that besides the two main genetic identities which are strictly required to form such patterns, a third population is likely to emerge, as a marker located at the interface between the two main identities. This prediction is then tested by dissecting petals of _Hibiscus trionum_ and performing an mRNA-seq survey. The resulting data set is consistent with the simulations, with a population of genes specifically expressed at the boundary between the two main regions. The paper then discusses a number of hypotheses on the evolution of underlying gene regulatory networks, testing them computationally. In particular, by comparing simulations with and without stochastic terms in the dynamics of gene regulation/expression, it is suggested that the 3rd identity is contributing to robustness of the pattern in the face of noise. Overall the main text is clear and makes an interesting case.

Major comments:**

1. The code used for simulations is available on a public repository, but it does not directly ensure that results are reproducible. To do so would require a clear step-by-step guide referring the user to the specific pieces of code which have been used for the results and figures presented in the paper. At the moment, I could not find any such guide and the large number of scripts, executables and jupyter notebooks are not clearly linked to the paper's contents.

2. The methods themselves involve a number of arbitrary choices. Though this is understandable given the nature of the work, one aspect in particular that would deserve better clarity is the modeling of gene network dynamics. The stochastic model (l.516 & following) involves a nesting of "Hill-like" terms (those in Eqs. (7) and (11)) which is unusual and given without justification. There should be some explanation of how this approach relates to standard approaches such as those reviewed e.g. in: Bintu et al. Current opinion in genetics & development 15.2 (2005): 116-124.

© 2026. Published by The Company of Biologists under the terms of the Creative Commons Attribution License (https://creativecommons.org/licenses/by/4.0/).

3. It is also unclear at the moment how exactly the GRN dynamics is used; are time-stepping algorithms used until the system reaches a stationary regime? If so, how is stationarity assessed? This needs to be explained both in the main text and in the methods. The table of parameters suggests that there was a cut-off time, but there is no explanation whatsoever about the state of the dynamics at this time.

4. Related to the previous point, the table of parameters (Table S1) is provided without any explanation; through what process (exploratory, literature review, trial and error...) where the values selected? As there been any type of sensitivity analysis?

Minor comment:

1. The fitness function used in simulations specifically encodes the desired pattern, with two zones having differential gene expression. This allows the artificial selection to evolve towards such patterns, as expected, but it is not entirely clear how this relates to natural selection itself. At the very start of the paper, the authors briefly review some possible sources of selective pressure for flowers to exhibit patterns such as bullseye, among others. None of the selective factors would likely act on the plants as a direct incentive for two regions, as specified in the cost function. Instead, one may expect a more high level criterion, such as "conspicuousness" for a pollinator, for instance.
This is admittedly not naturally represented as a fitness function, but the choice of this function definitely influences the outcomes of a simulation.
Some further numerical experiments may allow to demonstrate that the exact cost function is not critical for the findings of the paper, but I understand they would likely be computationally costly, to the point of unfeasibility. This limitation should be mentioned at least.
2. [optional suggestion]
The number of genes used in the simulations is very small in comparison to real organisms. This is clearly justified by the complexity of the work, but one wonders if simulations could be made more efficient by using a much simplified approach for the gene network dynamics. At the time scales of interest, it seems that the use of SDEs and the numerical intricacies they require might be an unnecessary burden. Have the authors considered a much simpler approach, for instance based on Boolean models? Since the study only uses static tissues, all the GRN dynamics could be by-passed, determining steady states very quickly and using them to determine fitness. If this saved significant computational time, this would allow a more comprehensive survey of the "purely genetic" part of the model.

Referee cross-commenting

I agree with both other reviewers. As mentioned by them, our reviews bring complementary suggestions, while being overall in good agreement.

Significance

Reviewer's expertise: mathematical modeling, mathematical biology.

This paper is mostly a conceptual study, in which the majority of results are based on computer simulations. The findings are biologically interesting, but it is hard to prove these evolutionary claims through physical experiments. The complexity of the simulations requires a large number of technical assumptions and parameter choices, which overall make it very difficult to assess how plausible these simulations are, compared to the natural processes they are meant to represent. All the findings are well-argued and provide an overall convincing case, but it is by design impossible to fully assess experimentally. As such, this work will be mostly valuable to theoretical biologists, computational modelers, and researchers interested in "artificial life" and gene evolution.

Reviewer 2:

Evidence, reproducibility and clarity

In the manuscript entitled "Recurrent emergence of boundary cell types during evolution of floral bullseye patterns" Oud et al use computational modeling to determine how gene regulatory

© 2026. Published by The Company of Biologists under the terms of the Creative Commons Attribution License (https://creativecommons.org/licenses/by/4.0/).

networks can set up the prepattern for a bullseye pigmentation. They use a modeling template that is similar to the hibiscus petal primordium, create a gene regulatory network composed of the interaction of cell autonomous transcription factors, transcription factors that can diffuse from one cell to another, and cell-cell communication signals. Each simulation started from a diffusing signal from the base and all other genes with no expression. Such a signal diffusing from the base of the organ has been hypothesized many times in plant morphogenesis, so this is plausible. They started 35 populations with initially random GRNs and let them evolve for 30,000 generations selecting for simulated petals with higher bullseye fitness in each generation. All 35 generated bullseyes. The authors used a UMAP dimensionality reduction similar to single cell RNA-seq to identify different cell types in the models. I have not seen this analysis applied to modeling before, and I thought this approach was innovative. Interestingly 26 out of the 35 initiated a boundary cell type to help in the robust establishment of the symmetric bullseye, whereas 9 did not. There are two major ways these boundary cell types is established: (1) boundary specific gene expression and (2) two nested proximal genes with one extending beyond the other. Then the authors examine real hibiscus petals and identify boundary cells, which express 30 boundary specific genes. The authors then examine one of the GRNs from one of their populations and find that gene 5 is crucial for setting up the boundary. Finally, the analyze over evolutionary time in each population and see that these boundary cells come and go in the lineages, but they have a longer persistence time when there is noise in the modeling, suggesting that they add robustness to the generation of the bullseye.

Major comments:

There is a major missed opportunity to analyze the evolved networks. Only one of the 30 GRNs is analyzed in figure 4. Please add further analysis of the GRNs from all the populations. Within a population after 30K generations, how much variation is there in the GRNs of individuals? How similar are the optimal fitness evolved GRNs across all 35 populations? Are there common motifs across networks? Is there always an antagonism between proximal and distal proteins somewhere in the network? A lot of previous work on GRNs has established the function of common motifs, and these should be analyzed. Please provide all 30 gene regulatory networks in the supplement.

The purpose and significance of examining the evolutionary lineage is not clear. Please explain your logic. This is most important for Figure 5 where it becomes clear that the boundary cells are often formed transiently in the evolution of the GRN. If this boundary cell type does not persist, how can it help the petal generate a bullseye. What happens after the boundary cell type is lost? Has the GRN evolved into a more stable place where it no longer needs the boundary? In several instances it looks like they come and go many times. Please explain how these transient boundary cells in the evolutionary lineage can make a difference. This point also comes up in lines 113-115 "For each simulation, we traced back the ancestral lineage of the final fittest individual and sampled 12 of its ancestors at evenly spaced generational intervals, performing this analysis on each sampled ancestor." I could understand if the boundary cell type were developmentally transient, but I have a hard time what its significance is since it is evolutionarily transient.

It is worth saying more about how the 9 lineages without a boundary cell types manage to make a robust bull's eye pattern because this is also interesting.

How were 12 proteins chosen for the network, as opposed to 6 or 20 for instance? In the network pruning, it seems like fewer proteins are required. How many proteins are required to produce a bulls eye pattern?

Minor comments:

The title needs to be changed to include computational modeling or simulation because otherwise the current version of the title implies that these boundary cell types are found in plant species evolution.

Line 103 - 106 "We found that over a third of all simulations evolved a bullseye size of approximately 50% of the petal's central height (Figure 2A.ii). This indicates a tendency for simulations to converge toward these proportions, possibly due to the interaction between the patterning signal distribution and the tissue geometry." The phrasing here is confusing. Which proportions does "these proportions" refer to? Presumably, 50% from the preceding sentence.

© 2026. Published by The Company of Biologists under the terms of the Creative Commons Attribution License (https://creativecommons.org/licenses/by/4.0/).

But the second proportion is not clear from the text. Maybe it is the peak at approximately 65% seen in the graph. Please clarify in the text.

Line 118 "To further explore cell identity in the third cluster, we analysed the gene expression profiles of the three identified cell types." It is not clear what the third cluster refers to. The previous sentence mentions 9 lineages without boundary cell types. So, a transition here back to lineages with boundary cell types, would help here.

Figures 3C-D, it would help to label these volcano plots proximal versus boundary and distal versus boundary. Although they do fit your color scheme and legend for the color scheme, it is important to specify it explicitly.

On Figure 4A it would help to label which gene is Prox and Dist. I assume they are the purple and yellow genes, but it would be easier if they were labeled.

Line 185-186 "Gene 5 delays and spatially restricts the expression of gene 10, ensuring the symmetric development of the pattern." This statement needs to be supported by showing a time series simulation-movie or timepoints-revealing this timing aspect of Gene 5.

Referee cross-commenting

I agree with all reviews, which are aligned.

Significance

How pigment patterns in petals are established is an important and fascinating question, that sheds light on broader issues of how tissues are pre-patterned. Previous studies focus on the reaction diffusion gene regulatory networks that create beautiful petal pigment spot patterns. This paper fills the gap in addressing how a prepattern is established to create a simple proximal distal bullseye pigment pattern. Overall, the use of modeling in this study raises several novel and exciting hypotheses for how a pre-pattern can be established during development. One limitation of the study as acknowledged by the authors is that the actual petal grows, whereas the model does not. Although growth is likely to make an interesting contribution to the pattern, I agree that it is beyond the scope of this manuscript. Modeling papers are always challenging to write clearly, and I point out some areas where clarifications are needed below. The figures illustrate the results well.
This paper will be of interest to developmental biologists, gene regulatory network afficionados and computational biologists.

My expertise is in plant morphogenesis and patterning as well as computational modeling.

Reviewer 3:

Evidence, reproducibility and clarity

The manuscript by Oud et al. explores the evolution of a developmental mechanism generating bullseye patterns in petals using evolutionary simulations of gene regulatory networks and transcriptomics data. The authors provide a plausible mechanism of how a novel cell type can emerge as a by product of selecting for a higher-order process-in this case, the establishment of a bullseye pattern with two clearly delineated regions. Moreover, the authors show that the emergence of the new cell type persists longer in their evolutionary simulations when the system is noisy, suggesting a functional role of the cell type in buffering developmental variability. The approach is very impressive, bridging in silico-generated GRNs that model a patterning process and evolve over generations, and in turn, combining them with transcriptome analysis experiments. However, precisely due to the complexity of the work done, I would like the authors to clarify and/or address key elements of the methodology, especially those related to the assumptions regarding the modelling approach and their implications for the validity of the results, as well as from the analysis.

© 2026. Published by The Company of Biologists under the terms of the Creative Commons Attribution License (https://creativecommons.org/licenses/by/4.0/).

Major comments:

1. There are some aspects to clarify; some are mentioned here, but others are mentioned in minor points.

1.1. How are the cell types defined from the simulations? Are they attractors of the dynamics of the corresponding proteins? And how are they computationally defined? Please provide more details about how the HBSCAN was used.
In Figure S5, simulations #6 and #8 appear to have a 4th cell type (coloured in green), but the authors do not mention this result in the text. If cell types are defined by gene expression profiles, then the number of cell types will be dependent on the kind of clustering performed.
Clarifying the definition of cell types will help resolve this issue.

1.2. In relation to the previous question, are the phenotypes used in the evolutionary simulations' steady states of the underlying dynamics?

1.3. In Figure 3A it seems there are probably two cell types in the boundary region, is that right? Or are the elongated purple and elongated white cells basically the same cell type?
Please clarify. If there are two, why did the authors choose to do the transcriptome analysis of the boundary region as one region, and not two subregions, to capture the two cell types?

1.4. I appreciate the explanation of the GRN pruning in the methods, but could the authors illustrate the network pruning process with an example and show that it works in this example?

1.5. From the methodological perspective, I suggest further clarifying what is new from this study and what is not. For instance, is the GRN pruning idea new or has it done before? The authors could consider reducing the formalities in the methods of the main text when they are not needed or when they are not new, to facilitate the readability of what is really important and novel in this work, and what is not. E.g., it is not really needed to mathematically define a Voronoi tessellation in the main methods section; this could be simplified or moved to a supplementary methods section.
2. I believe the diffusion term used in Eqs. 14 and 17 does not conserve the total number of protein molecules; could the authors verify that? An example of a correct passive transport term for cell i of protein concentration p_i would be the sum of (p_j-p_i) for all j-cell neighbours, normalized by the area of cell i, or the formulation by Sukumar and Bolander (2003). This is especially important when noise is added, as the non-conservation of the number of proteins can lead to unwanted instabilities. Likely, these effects do not invalidate the results of the paper, but the authors should clarify the reason for their choice or double- check the conclusions using a correct, mass-conserved diffusion term.
3. It is important to facilitate the reproducibility of the results whenever possible, especially given that the computational framework used in this work has great value. I truly appreciate that the authors uploaded the code to a Gitlab. Please add further information in the readme file to facilitate reproducing the results, beyond the information regarding the code installation, whenever possible.

Minor comments:

4. What is the reasoning behind the choice of the number of protein species? Why 12?
Would the same results hold with a smaller number of proteins? As I imagine that the more species one considers, the more chances one has to get the desired phenotypes (or any desired phenotype for that matter). I could imagine that with 12 or more proteins, one could get more than 3 cell types (as defined by the clustering of their expression profiles). Is there something inherent in the creation of a boundary that leads to only 1 additional cell type and not more? Further simulations would be ideal to address this point, but otherwise, please comment on that if possible.
5. What is the fundamental difference between Gene profiles I and II in generating celltypes? If a cell type is defined by the specific expression of certain genes, then are not GeneProfiles I and II just different sides of the same coin? For instance, Gene profile I is characterized by the expression of a single gene at the boundary. Why do their simulations they do not obtain patterns where 2 genes are expressed in the boundary? Or 3? Or is there a fundamental difference in how these are generated, like the boundary being a stripe of a Turing pattern, or something similar? This also links

© 2026. Published by The Company of Biologists under the terms of the Creative Commons Attribution License (https://creativecommons.org/licenses/by/4.0/).

with the work of Ding et al. and Lu et al.-which the authors mention in the introduction- where they propose that self-organized (Turing) patterns can explain anthocyanin patterning in petals. Could the authors clarify these points and may be contextualize these results with previous works on petal patterns?
6. In relation to the previous point regarding the mechanisms underlying boundary formation,the authors could consider whether the theoretical works by the J. Sharpe lab on stripe formation might be relevant to cite (e.g., Cotterell and Sharpe 2010 or Jimenez et al 2015)
7. If possible, it would be ideal to have at least one video/animation of both the dynamics of each phenotype and the evolution of the phenotypes as their fitness increases, to see the evolutionary trajectories and test whether similar phenotypes can be achieved through different trajectories.
8. In the Discussion, I believe that the emergence of the novel cell type would benefit from stronger contextualization within known evo-devo frameworks. In particular, the authors describe that a new cell type emerges as a by product of the selection of a higher-order developmental process-the bullseye pattern with a clearly defined boundary-rather than through direct selection of the cell type itself. I am confident the authors know these phenomena have been discussed under the term spandrels (Gould & Lewontin, 1979), and have been the subject of extensive study and debate. While identifying traits as spandrels is complicated-largely because in practice we lack reliable frame works to distinguish them from actual adaptations-the work presented here provides a plausible mechanism of how such features could arise. To me, this fact alone is interesting, as not many works (as far as I know) have addressed this problem explicitly. Maybe the authors want to emphasize this fact as a novelty of their approach. To be clear, I am not suggesting that the authors should adopt a specific terminology; rather, I believe that explicitly invoking the concept of spandrel would resonate with readers familiar with the foundations of evo-devo and would strengthen the main message of the paper.
9. Some additional considerations related to figures:
9.1. Please change colours in the figures to be colour-blind whenever possible.
9.2. The stripes in the striped purple cell shown in Fig. 3A are not seen unless one zooms in on it; would it be possible to represent this differently?
9.3. In Fig. 5 Aii and Bii, it would be easier for the reader to connect with the statements in the main text if the x-axis is x 1000 or x100 instead of x500
9.4. Perhaps clarify panel captions of Fig. panels 3C and 3D. Probably I am missing something basic, but I was also wondering how their numbers are connected to the numbers in the panel of Fig. 3F.
9.5. Why does Fig. 3F have three subpanels? Is it because of different expression levels?
Please clarify.
10. Could the authors clarify the choice of using the Stratonovich approach in the stochastic simulations?
11. Note equations are referred to in the text as Eq. S (...) whereas they are not supplementary equations.
12. The code is very large (more than 1GB), and I believe much of the space is used by Voronoi tessellations. If the authors have the time and have the scripts generating theVoronoi tessellations, the authors could add them to the repository and ensure that these tessellations are generated during the simulations whenever needed (but I am aware that code organization takes time). I would recommend having the code also in a repository with a DOI (e.g., Zenodo or OSF).

Referee cross-commenting
The comments by other referees are complementary to mine; there are some common aspects with my comments and other important points to look into.

Significance

This study provides a plausible explanation of how new cell types can emerge as by products of the selection of other processes. This is an important advance in understanding the mechanisms underlying the origin of evolutionary novelties, particularly from the point of view of morphogenesis and patterning, rather than from a more traditional, strictly gene-centric views which focus on changes in specific loci, gene duplications, or neofunctionalization. By highlighting evolutionary novelty as a consequence of higher-order constraints, this work broadens the frameworks through which cellular diversity can be understood.

I believe most of the limitations of the study are conceptual and regarding improving clarity rather than methodological. For instance, the definition of what a cell type is remains, in my

© 2026. Published by The Company of Biologists under the terms of the Creative Commons Attribution License (https://creativecommons.org/licenses/by/4.0/).

opinion, somewhat vague, especially if the clustering has been performed with only 12 genes. However, I am aware of the conceptual difficulty in defining cell types in general. In addition, the emergence of only a single additional cell type, rather than multiple types, might be a consequence of the limited number of proteins considered. Aside from these issues, the methodology is sound and provides a useful framework for exploring the origin of novel cell types.

I see this work as being of substantial interest to researchers concerned with the conceptual foundations of evo-devo, particularly those interested in the origins of novelty and in the role of constraints in shaping such novelty. It should also be relevant to studying morphogenesis from a dynamical systems perspective. Finally, this work will be of interest to those investigating the ecological roles of petal patterns, especially in relation to their roles in attracting pollinators or protecting reproductive organs from environmental factors.

Overall, I think this work represents a very valuable contribution to the evo-devo community, providing conceptual advances into our understanding of the emergence of novelty, as well as providing a complex computational framework addressing cellular patterning in evolving GRNs.

Field of expertise: developmental biology, nonlinear dynamics, pattern formation, evo-devo.

Author response to reviewers' comments

Manuscript number: RC-2025-03323
Corresponding author(s): Steven, Oud

[Please use this template only if the submitted manuscript should be considered by the affiliate journal as a full revision in response to the points raised by the reviewers.

If you wish to submit a preliminary revision with a revision plan, please use our "Revision Plan" template. ***It is important to use the appropriate template to clearly inform the editors of your intentions.]***

1. General Statements [optional]

We thank the reviewers for their extensive and constructive feedback, which has led to significant modifications that we feel have greatly improved the manuscript. Based on their suggestions, we performed additional analyses, including a closer examination of motifs within the gene regulatory networks and a more detailed characterisation of non-boundary-forming patterning dynamics. We also addressed common feedback across reviewers regarding the clarity of our simulation procedures by improving the description of our implementation in the methods section and by adding supplementary figures. Finally, we have made our software more accessible by providing all analysis scripts and improving the accompanying documentation in the repository. In the revision document below, we address all reviewers' concerns point by point. We look forward to your evaluation of our revised manuscript.

2. Point-by-point description of the revisions

**Reviewer 1**

© 2026. Published by The Company of Biologists under the terms of the Creative Commons Attribution License (https://creativecommons.org/licenses/by/4.0/).

1 . The code used for simulations is available on a public repository, but it does not directly ensure that results are reproducible. To do so would require a clear step-by-step guide referring the user to the specific pieces of code which have been used for the results and figures presented in the paper. At the moment, I could not find any such guide and the large number of scripts, executables and jupyter notebooks are not clearly linked to the paper's contents
We agree that the code should be as accessible as possible for reproducing the results. We have updated the public repository (linked given in the 'data and code availability' section of the manuscript, lines 350-352) to include the SLURM job scripts used to run the evolutionary simulations and analyses, together with an overview of which scripts and notebooks were used for creating the figures.
2 . The methods themselves involve a number of arbitrary choices. Though this is understandable given the nature of the work, one aspect in particular that would deserve better clarity is the modeling of gene network dynamics. The stochastic model (l.516 & following) involves a nesting of "Hill-like" terms (those in Eqs. (7) and (11)) which is unusual and given without justification. There should be some explanation of how this approach relates to standard approaches such as those reviewed e.g. in: Bintu et al. Current opinion in genetics & development 15.2 (2005): 116-124.
We agree that the formulation of the developmental model requires clearer justification and contextualisation. We have added a citation to situate our implementation within existing modelling frameworks, and a brief explanation of the choice for Hill equations in the Methods section (lines 577-579).
3. It is also unclear at the moment how exactly the GRN dynamics is used; are time-stepping algorithms used until the system reaches a stationary regime? If so, how is stationarity assessed? This needs to be explained both in the main text and in the methods. The table of parameters suggests that there was a cut-off time, but there is no explanation whatsoever about the state of the dynamics at this time.

© 2026. Published by The Company of Biologists under the terms of the Creative Commons Attribution License (https://creativecommons.org/licenses/by/4.0/).

We have revised the main text to briefly explain how the developmental dynamics are implemented (lines 88-90) and expanded the Methods section (Gene expression and regulation in the developmental model) to describe the integration procedure in detail (lines 617-620).

The GRN dynamics are modelled as stochastic differential equations (SDEs), which are numerically integrated for a fixed developmental duration of T_D = 140 hours, regardless of whether a stationary state is reached.

Instead, stationarity is indirectly favored by the fitness function. Fitness is calculated as the time average of the phenotype (protein states) over a window at the end of development (Equation 23 in the Methods). As a result, GRNs that exhibit large fluctuations or ongoing transient dynamics during this evaluation window tend to have lower fitness (and in turn, reproduction rate) than GRNs that have stabilised their expression patterns. We now mention this in the model introduction of the results section (lines 98-99).

As a result of this, we observe that the vast majority of evolved GRNs reach a stable gene expression state by the end of development (aside from small fluctuations as expected from the SDEs).

4 . Related to the previous point, the table of parameters (Table S1) is provided without any explanation; through what process (exploratory, literature review, trial and error...) where the values selected? As there been any type of sensitivity analysis?

We have clarified in the revised manuscript how each group of parameters was chosen (lines 618-620 and 744-746). In brief:

- Developmental time parameters (e.g., integration time, diffusion coefficient) were set to roughly match the developmental window of *H. trionum* from stage 0 to stage 2 (~150 hours; Riglet et al. 2024), during which pre-patterning is established.
- Molecular concentrations are expressed in arbitrary units
- Evolutionary parameters (e.g., mutation rates) are based on previous published work using this modeling framework and were slightly adjusted during an initial exploratory phase to ensure stable evolutionary dynamics. We have added citations for this.

We have not performed a full global sensitivity analysis across all parameters. Such an analysis would be computationally expensive given the cost of running evolutionary simulations and the difficulty of assessing parameter effects in this multi-scale system. Importantly, the core GRN parameters (expression rates, interaction topology, and interaction strengths) are evolvable rather than fixed. We have conducted sensitivity analyses at the level of individual evolved GRNs, but a systematic analysis is beyond the scope of this paper.

Minor Comments

© 2026. Published by The Company of Biologists under the terms of the Creative Commons Attribution License (https://creativecommons.org/licenses/by/4.0/).

1 . *The fitness function used in simulations specifically encodes the desired pattern, with two zones having differential gene expression. This allows the artificial selection to evolve towards such patterns, as expected, but it is not entirely clear how this relates to natural selection itself.*<br>*At the very start of the paper, the authors briefly review some possible sources of selective pressure for flowers to exhibit patterns such as bullseye, among others. None of the selective factors would likely act on the plants as a direct incentive for two regions, as specified in the cost function. Instead, one may expect a more high level criterion, such as "conspicuousness" for a pollinator, for instance.*<br>*This is admittedly not naturally represented as a fitness function, but the choice of this function definitely influences the outcomes of a simulation.*<br>*Some further numerical experiments may allow to demonstrate that the exact cost function is not critical for the findings of the paper, but I understand they would likely be computationally costly, to the point of unfeasibility. This limitation should be mentioned at least.*
We agree that natural selection acts on higher-level criteria such as pollinator attraction or conspicuousness rather than a predefined measure like “two distinct regions.” However, our goal in this study is specifically to understand how the bullseye pattern in particular is produced, motivated by comparison to *Hibiscus* and other angiosperms where this pattern has documented adaptive relevance. The fitness function was therefore designed to ensure this particular pattern evolved, which results in evolving between-level novelty rather than constructive novelty (as defined in Colizzi *et al.*, Essays Biochem 2022: of interest here is the evolved dynamics of development, not the resulting pattern). In this way, the fitness function serves as a proxy for selection on floral patterning. We have clarified this rationale more explicitly in the Results section (lines 97-98).<br><br>The choice of fitness function does influence simulation outcomes. Within the scope of selecting for a bullseye pattern, we previously ran simulations where bullseye size was fixed rather than dynamic, and boundary cell types still evolved in those cases. This suggests our findings are robust across variations of the bullseye fitness function. Of course, selecting for a more abstract ecological criterion such as “conspicuousness” rather than a distinct spatial pattern would affect outcomes more substantially. However, translating such high-level criterion into a quantitative fitness function is a non-trivial challenge and outside the scope of this study. We have added a note on this point in the Methods section on the fitness function (lines 687-691).
2 . *The number of genes used in the simulations is very small in comparison to real organisms. This is clearly justified by the complexity of the work, but one wonders if simulations could be made more efficient by using a much simplified approach for the gene network dynamics. At the time scales of interest, it seems that the use of SDEs and the numerical intricacies they require might be an unnecessary burden. Have the authors considered a much simpler approach, for instance based on Boolean models? Since the study only uses static tissues, all the GRN dynamics could be by-passed, determining steady states very quickly and using them to determine fitness. If this saved significant computational time, this would allow a more comprehensive survey of the "purely genetic" part of the model.*

© 2026. Published by The Company of Biologists under the terms of the Creative Commons Attribution License (https://creativecommons.org/licenses/by/4.0/).

While the number of genes may indeed be indeed small compared to real organisms, our simulations should be viewed as operating on subnetworks that form part of a much larger developmental GRN. This is a common approach in modelling the evolution of developmental processes, which we now highlight in the methods section. Furthermore, we find that the functional part of the GRN (which we identify by pruning away the redundant genes and interactions) always uses only a subset of the gene types, showing that we provide sufficient degrees of freedom for the evolutionary process to find a solution. We now also make note of this in a new figure (Figure S12) where we explain the pruning algorithm.

We agree that simplified representations of the GRN, such as Boolean models or direct steady-state mappings, could substantially reduce computational cost. However, the use of stochastic differential equations (SDEs) in the present study is deliberate. Continuous, stochastic GRN dynamics allow us to capture key features that would be difficult or impossible to represent in Boolean or purely steady-state frameworks. In particular, they enable (i) gradual spatial distributions of morphogens, which are central to pattern formation,

(ii) explicit treatment of gene expression noise, and (iii) consider and analyse the developmental dynamics in detail.
Finally, in response to Reviewer 2’s comment 1, we show all evolved networks (Figure S3 & S4) and perform a GRN motif comparison between noisy and deterministic simulations (Figure S15) to provide more information about the genetic part of the model.

Reviewer 2

1 . There is a major missed opportunity to analyze the evolved networks. Only one of the 30 GRNs is analyzed in figure 4. Please add further analysis of the GRNs from all the populations. Within a population after 30K generations, how much variation is there in the GRNs of individuals? How similar are the optimal fitness evolved GRNs across all 35 populations? Are there common motifs across networks? Is there always an antagonism between proximal and distal proteins somewhere in the network? A lot of previous work on GRNs has established the function of common motifs, and these should be analyzed. Please provide all 30 gene regulatory networks in the supplement.

© 2026. Published by The Company of Biologists under the terms of the Creative Commons Attribution License (https://creativecommons.org/licenses/by/4.0/).

We have substantially expanded the analysis of evolved networks across all populations. Specifically, we now (i) provide two supplementary figures showing the final pruned GRNs from all 35 simulations (Figures S3 & S4), and (ii) quantify motif frequencies across all evolved networks and compare motif distributions between GRNs evolved with and without molecular noise (Figure S15). This new analysis is summarised in a dedicated Results paragraph where we identify regulatory asymmetries and condition-dependent differences in feedback architecture, including changes in abundance of mutual inhibition and positive autoregulation (lines 233-239).<br><br>We find that, while the evolved maximum fitnesses are very similar across simulations (Fig. 2Ai), the networks are highly variable. Nevertheless, the motif analysis shows some trends that differ between the noise and no-noise simulations, such as a bias towards mutual inhibition between PROX and DIST in the no-noise compared to the noise simulations.<br>As to the variation within a population: we find that at any timepoint, all individuals are descended from a common ancestor that lived on average ~600 generations back, meaning that they form a single (quasi)species. We therefore analyse a single, highly fit individual at the last timepoint.
2 .   *The purpose and significance of examining the evolutionary lineage is not clear. Please explain your logic. This is most important for Figure 5 where it becomes clear that the boundary cells are often formed transiently in the evolution of the GRN. If this boundary cell type does not persist, how can it help the petal generate a bullseye.*
What happens after the boundary cell type is lost? Has the GRN evolved into a more stable place where it no longer needs the boundary? In several instances it looks like they come and go many times. Please explain how these transient boundary cells in the evolutionary lineage can make a difference. This point also comes up in lines 113-115 "For each simulation, we traced back the ancestral lineage of the final fittest individual and sampled 12 of its ancestors at evenly spaced generational intervals, performing this analysis on each sampled ancestor." I could understand if the boundary cell type were developmentally transient, but I have a hard time what its significance is since it is evolutionarily transient.

© 2026. Published by The Company of Biologists under the terms of the Creative Commons Attribution License (https://creativecommons.org/licenses/by/4.0/).

The persistence of the boundary cell type over evolutionary time is used as a signal for its functional role in establishing the bullseye pattern. We observe that mostly two extremes occur: boundary cell types can be conserved over long evolutionary periods, or they can be highly transient. In our simulations, boundary cell types that are functionally important tend to persist, whereas the ones that are not involved in producing the bullseye pattern appear only transiently. The fact that both cases can occur suggests that boundary cell types are a "free" or easily accessible feature during the evolution of this patterning system: they can arise repeatedly without being strictly required, but may nonetheless become functionalised under certain evolutionary trajectories (see also our discussion of the *Mimulus* leaf stripe). We have added more explanation on the logic of examining the evolutionary lineage at the beginning of the results section related to Figure 5 (lines 205-209 and caption of Figure 5).

To further clarify this point, we have added a supplementary figure (Figure S16) focusing on a deterministic simulation with a highly evolutionarily transient boundary cell type. By identifying the GRN mutations associated with the (re-)appearance of the boundary, we show that the patterning mechanism producing the bullseye slowly mutates while preserving the bullseye, while the mutational neighbourhood of the GRN contains diverse mutations that generate boundary cell types. In this case, boundary cells arise independently through distinct mutations rather than repeated rediscovery of a single change, explaining both their frequent appearance and their lack of long-term evolutionary stability.

3 . It is worth saying more about how the 9 lineages without a boundary cell types manage to make a robust bull's eye pattern because this is also interesting.

This is indeed a good idea, we have carried out an analysis similar to that in Figure 4 for a GRN from a lineage without a boundary cell type and included it as a supplementary figure (Figure S11).

4 . How were 12 proteins chosen for the network, as opposed to 6 or 20 for instance? In the network pruning, it seems like fewer proteins are required. How many proteins are required to produce a bulls eye pattern?

This choice is indeed somewhat arbitrary. We settled on 12 gene types to provide enough degrees of freedom while also keeping the evolutionary simulations computationally feasible. In practice, we find that pruned GRNs typically only use a subset of the 12 gene types, suggesting that the system has enough degrees of freedom to produce the bullseye pattern. For example, the smallest networks that evolved (after pruning) have 5 genes in the deterministic model and 7 in the noisy model.

To clarify this choice, we now added a brief mention of these considerations to the relevant methods section (lines 641-643).

Minor Comments

1 . The title needs to be changed to include computational modeling or simulation because otherwise the current version of the title implies that these boundary cell types are found in plant species evolution.

© 2026. Published by The Company of Biologists under the terms of the Creative Commons Attribution License (https://creativecommons.org/licenses/by/4.0/).

We agree and have renamed the paper “Computational Model of Flower Pattern Evolution Predicts Spontaneous Emergence of Boundary Cell Types Across Petal Epidermis.”
2 . *Line 103 - 106 "We found that over a third of all simulations evolved a bullseye size of approximately 50% of the petal's central height (Figure 2A.ii). This indicates a tendency for simulations to converge toward these proportions, possibly due to the interaction between the patterning signal distribution and the tissue geometry." The phrasing here is confusing. Which proportions does "these proportions" refer to? Presumably, 50% from the preceding sentence. But the second proportion is not clear from the text. Maybe it is the peak at approximately 65% seen in the graph. Please clarify in the text.*
The 50% figure refers to the bin with the highest peak in Figure 2A.ii, reflecting a bias toward certain bullseye proportions rather than a uniform distribution across all possible sizes. We have rewritten the sentence to clarify this (lines 109-112): “This indicates a tendency for simulations to converge towards certain proportions more than others, possibly due to the interaction between the patterning signal distribution and the tissue geometry”
3 . *Line 118 "To further explore cell identity in the third cluster, we analysed the gene expression profiles of the three identified cell types." It is not clear what the third cluster refers to. The previous sentence mentions 9 lineages without boundary cell types. So, a transition here back to lineages with boundary cell types, would help here.*
We agree and have improved the phrasing here by referencing back to the lineages with boundary cell types (lines 124-125):<br>“Focusing on the majority of lineages in which this third boundary cell type arose, we analysed the gene expression profiles of the three identified cell types.”
4 . *Figures 3C-D, it would help to label these volcano plots proximal versus boundary and distal versus boundary. Although they do fit your color scheme and legend for the color scheme, it is important to specify it explicitly.*
We have added labels inside the volcano plots in Figure 3C-D to clarify proximal versus boundary and distal versus boundary.
5 . *On Figure 4A it would help to label which gene is Prox and Dist. I assume they are the purple and yellow genes, but it would be easier if they were labeled.*
We have added labels in Figure 4A here to clarify.

© 2026. Published by The Company of Biologists under the terms of the Creative Commons Attribution License (https://creativecommons.org/licenses/by/4.0/).

6 . Line 185-186 "Gene 5 delays and spatially restricts the expression of gene 10, ensuring the symmetric development of the pattern." This statement needs to be supported by showing a time series simulation-movie or timepoints-revealing this timing aspect of Gene 5.

We agree with the reviewer that this is currently lacking a clear visualisation and thank them for pointing this out. To address this, we have updated Figure 4 to include the temporal expression of genes 5 and 10 in the wild type and mutant for cells along the left-right axis in the proximal bullseye region. We have also included the following extra details in the results text (lines 194-199): Decreasing the spatial range of gene 5's regulatory influence by turning it into a TF resulted in a delay in its inhibition of gene 10 and reduced its self-activation range, explaining the smaller bullseye. In this mutant, expression of gene 5 is progressively delayed in cells located further from the origin of the patterning signal, and is ultimately absent on the right side of the proximal region of the bullseye (Figure 4C.ii). As a consequence, gene 10 becomes expressed in the right region, resulting in *DIST* identity instead of *PROX,* and leading to an asymmetric bullseye pattern.

Reviewer 3

1 . How are the cell types defined from the simulations? Are they attractors of the dynamics of the corresponding proteins? And how are they computationally defined? Please provide more details about how the HBSCAN was used. In Figure S5, simulations #6 and #8 appear to have a 4th cell type (coloured in green), but the authors do not mention this result in the text. If cell types are defined by gene expression profiles, then the number of cell types will be dependent on the kind of clustering performed. Clarifying the definition of cell types will help resolve this issue.

© 2026. Published by The Company of Biologists under the terms of the Creative Commons Attribution License (https://creativecommons.org/licenses/by/4.0/).

We thank the reviewer for raising this point and agree that the definition of cell types in our simulation results requires clearer explanation.

The concept of cell type / cell identity is a complex theme which is still yielding interesting debate and discussion in the literature (see for instance Rafelski and Theriot, 2024). In our simulations, cell types are defined based on gene expression profiles rather than being explicitly identified as mathematical attractors of the underlying dynamical system.
Operationally, we perform dimensionality reduction (UMAP) followed by clustering (using HDBSCAN) on the gene expression profiles across cells. This clustering serves as an initial, automated indication of distinct expression states across the petal.
We recognise that the clustering results depend on the chosen dimensionality reduction and clustering method, as well as their parameterisation. For example, clustering applied to a smooth gradient (e.g., arising from diffusion alone) can artificially partition continuous variation into multiple discrete groups. For this reason, we do not rely solely on the clustering output: we use it as a first-pass classification and then manually verify the resulting groups by manually inspecting their gene expression profiles across the petal. This additional step ensures that identified “cell types” correspond to distinct expression states rather than arbitrary thresholds along a gradient. We have clarified both the computational procedure (dimensionality reduction + HDBSCAN clustering + manual verification) and the conceptual definition of cell types in the Methods section (lines 748-753).
Regarding Figure S5, the fourth cell type (shown in green) in simulations #6 and #8 is indeed a distinct gene expression profile. We do occasionally observe the evolution of more and different cell types, this second boundary cell type being one of them, but also for example a salt-and-pepper type cell type (not shown). These cell types are however usually very transient and infrequent.

Rafelski, S.M. and Theriot, J.A., 2024. Establishing a conceptual framework for holistic cell states and state transitions. Cell, 187(11), pp.2633-2651.

2 . *In relation to the previous question, are the phenotypes used in the evolutionary simulations' steady states of the underlying dynamics?*

As clarified in response to Reviewer 1’s comment 3, we do not explicitly require or enforce that phenotypes correspond to steady states of the underlying GRN dynamics. The developmental dynamics are always simulated for a fixed duration, and the fitness of a GRN is defined as the time-averaged gene expression pattern over a window at the end of this (lines 88-90) and Methods (lines 617-620).
Because fitness is computed from this late-stage average, selection favors GRNs that produce consistent and stable expression patterns during that window. Networks that remain in strong transient or oscillatory regimes during this phase are typically penalised through reduced fitness. Therefore, while steady states are not imposed as a constraint, selection strongly favors solutions that are effectively stationary by the end of development. Indeed, inspection of the evolved GRNs shows that they converge to stable expression states.

© 2026. Published by The Company of Biologists under the terms of the Creative Commons Attribution License (https://creativecommons.org/licenses/by/4.0/).

3 . In Figure 3A it seems there are probably two cell types in the boundary region, is that right? Or are the elongated purple and elongated white cells basically the same cell type? Please clarify. If there are two, why did the authors choose to do the transcriptome analysis of the boundary region as one region, and not two subregions, to capture the two cell types?
Correct, there are two different boundary cell types at the mature stage 5 petal: flat, elongated purple cells (lower boundary), and flat, elongated cream cells (upper boundary). However, the transcriptome data comes from an earlier stage (stage 2), where the boundary cells have not yet developed their characteristic shape and texture and the petal only comprises visibly pigmented (proximal) and non-pigmented (distal) cells. The morphological differences that distinguish the two boundary cell types at stage 5 are not yet apparent, hence we can only treat the boundary as one region at this stage, defined as the transition zone between pigmented and unpigmented cells<br><br>We have made this distinction clearer in the figure caption of the Stage 2 petal (Figure 3B).
4 . I appreciate the explanation of the GRN pruning in the methods, but could the authors illustrate the network pruning process with an example and show that it works in this example?
We have added a supplementary figure (Figure S12) depicting the pruning process for a GRN which keeps its boundary cell type during pruning and one for a GRN which loses its boundary cell type after pruning.
5 . From the methodological perspective, I suggest further clarifying what is new from this study and what is not. For instance, is the GRN pruning idea new or has it done before? The authors could consider reducing the formalities in the methods of the main text when they are not needed or when they are not new, to facilitate the readability of what is really important and novel in this work, and what is not. E.g., it is not really needed to mathematically define a Voronoi tessellation in the main methods section; this could be simplified or moved to a supplementary methods section.
We agree that the distinction between methodological novelty and established components of the framework should be made clearer. We have therefore streamlined the description of non-novel methods and added appropriate citations to prior work where relevant, for example in the section on pruning.
6 . I believe the diffusion term used in Eqs. 14 and 17 does not conserve the total number of protein molecules; could the authors verify that? An example of a correct passive transport term for cell i of protein concentration p_i would be the sum of (p_j-p_i) for all j-cell neighbours, normalized by the area of cell i, or the formulation by Sukumar and Bolander (2003). This is especially important when noise is added, as the non-conservation of the number of proteins can lead to unwanted instabilities.<br>Likely, these effects do not invalidate the results of the paper, but the authors should clarify the reason for their choice or double-check the conclusions using a correct, mass-conserved diffusion term.

© 2026. Published by The Company of Biologists under the terms of the Creative Commons Attribution License (https://creativecommons.org/licenses/by/4.0/).

Thank you for pointing this out, this is indeed an error in our mathematical description. We double-checked our implementation, and confirmed our implementation correctly normalises by the area of cell i. We have a unit test which tests for mass conservation (https://gitlab.developers.cam.ac.uk/slcu/teamrv/evo-framework/-/blob/paper-2024-stoch-sims/tests/petal_test.cc?ref_type=tags#L66), which also confirms that our implementation is correct and this is only an error in the mathematical description in the paper. We have updated the equations to correctly reflect the implementation.

7 . It is important to facilitate the reproducibility of the results whenever possible, especially given that the computational framework used in this work has great value. I truly appreciate that the authors uploaded the code to a Gitlab. Please add further information in the readme file to facilitate reproducing the results, beyond the information regarding the code installation, whenever possible.

We thank the reviewer for emphasising the importance of reproducibility. As noted in our response to Reviewer 1's comment 1, we have improved the structure and documentation of the public repository to facilitate reproduction of the results, including the SLURM scripts used for the evolutionary simulations and documenting code used for analysis and creating figures.

Minor Comments

1 . What is the reasoning behind the choice of the number of protein species? Why 12? Would the same results hold with a smaller number of proteins? As I imagine that the more species one considers, the more chances one has to get the desired phenotypes (or any desired phenotype for that matter). I could imagine that with 12 or more proteins, one could get more than 3 cell types (as defined by the clustering of their expression profiles). Is there something inherent in the creation of a boundary that leads to only 1 additional cell type and not more? Further simulations would be ideal to address this point, but otherwise, please comment on that if possible.

As noted in our response to Reviewer 2's comment 4, the choice of 12 protein species is to some extent arbitrary. We selected this number as a compromise between providing sufficient degrees of freedom and maintaining computational feasibility of the evolutionary simulations. In a recently published manuscript from our team (van der Jagt et al., 2026), we tested the impact of reducing the number of genes and showed that important evolutionary dynamics are by and large the same.

Regarding the possibility of obtaining more than three cell types: while rare, we do observe the emergence of additional cell types in simulation #6 and #8 in Figure S9. A larger number of proteins could in principle support more combinations of expression patterns, but the number of stable cell types that emerge is strongly determined by the fitness function and by the spatial structure of the task (i.e., generating two pre-specified domains). That is, the emergence of a single additional boundary cell type is driven primarily by the developmental and selective constraints, rather than being directly limited by the number of proteins in our simulations.

van der Jagt, Pjotr L., Steven Oud, and Renske MA Vroomans. "System drift in the evolution of plant meristem development." PLOS Genetics 22.4 (2026): e1012089.

© 2026. Published by The Company of Biologists under the terms of the Creative Commons Attribution License (https://creativecommons.org/licenses/by/4.0/).

What is the fundamental difference between Gene profiles I and II in generating cell types? If a cell type is defined by the specific expression of certain genes, then are not Gene Profiles I and II just different sides of the same coin? For instance, Gene profile I is characterized by the expression of a single gene at the boundary. Why do their simulations they do not obtain patterns where 2 genes are expressed in the boundary? Or 3? Or is there a fundamental difference in how these are generated, like the boundary being a stripe of a Turing pattern, or something similar? This also links with the work of Ding et al. and Lu et al.-which the authors mention in the introduction- where they propose that self-organized (Turing) patterns can explain anthocyanin patterning in petals. Could the authors clarify these points and maybe contextualize these results with previous works on petal patterns?

The fundamental difference between the two gene profiles lies in how the boundary cell type is generated. In gene profile II, genes expressed in the boundary are also expressed in the proximal region, but some genes expressed proximally are not present in the boundary. The boundary cell type therefore emerges as the intersection of two differently-sized proximal bullseyes (Fig. 2B.ii). In gene profile I, by contrast, genes are more expressed in the boundary than anywhere else, producing a central striped expression pattern. While gene profile I can arise from profile II (Fig. S10), we also find cases where mechanism I appears independently, without mechanism II being present (Fig. S9; Simulation #25). This shows the two mechanisms are genuinely distinct, and we therefore treat them separately.

Profile I includes infrequent cases where several genes are preferentially expressed at the boundary (see for example simulation #23 in Figure S9). As for why we rarely observe two or more genes uniquely expressed in the boundary, we are not sure, however we suspect this may relate to the limited number of distinct gene types available in our model, which constrains how many genes can play a flexible, boundary-specific role.

Regarding the link to Turing patterns and the work of Ding et al. and Lu et al.: our model addresses the pre-patterning mechanism upstream of anthocyanin patterning, which subdivides the petal into distinct spatial regions. Based on evidence from *Hibiscus*, this pre-patterning is thought to be initiated by an asymmetric signal. The problem we investigate is therefore how an existing asymmetric signal is converted into a bullseye pattern, which is fundamentally different from Turing-type symmetry breaking from a uniform state. Our work thus complements Ding et al. and Lu et al. by addressing the upstream question of how the spatial regions that constrain these self-organised patterns to specific petal domains are first established. We have added a discussion of this connection in the Discussion section (lines 301-306).

3 . In relation to the previous point regarding the mechanisms underlying boundary formation, the authors could consider whether the theoretical works by the J. Sharpe lab on stripe formation might be relevant to cite (e.g., Cotterell and Sharpe 2010 or Jimenez et al 2015)

We agree that they are relevant and have added a section about theoretical work on stripe formation as part of the discussion on novel phenotypes (lines 305-310).

4 . If possible, it would be ideal to have at least one video/animation of both the dynamics of each phenotype and the evolution of the phenotypes as their fitness increases, to see the evolutionary trajectories and test whether similar phenotypes can be achieved through different trajectories.

© 2026. Published by The Company of Biologists under the terms of the Creative Commons Attribution License (https://creativecommons.org/licenses/by/4.0/).

We thank the reviewer for the suggestion, since the temporal dynamics can indeed be informative. We have added two supplementary videos (Video S1 & S2) illustrating the developmental dynamics of two GRNs: one that generates a boundary cell type via gene profile I, and one via gene profile II. These videos provide a clearer view of the developmental model's dynamics, and how boundary cell types emerge dynamically during development. References to these videos have been added to the main text immediately after introducing the two gene profiles.

In addition, we have added two supplementary figures containing evolutionary trajectories: one tracing an individual's evolutionary trajectory including detailed changes in fitness and gene expression over time (Figure S8), and one showing the evolution of *PROX* and *DIST* expression during the early adaptive phase across the first 10 simulations (Figure S6).

5 . *In the Discussion, I believe that the emergence of the novel cell type would benefit from stronger contextualization within known evo-devo frameworks. In particular, the authors describe that a new cell type emerges as a byproduct of the selection of a higher-order developmental process-the bullseye pattern with a clearly defined boundary-rather than through direct selection of the cell type itself. I am confident the authors know these phenomena have been discussed under the term spandrels (Gould & Lewontin, 1979), and have been the subject of extensive study and debate. While identifying traits as spandrels is complicated-largely because in practice we lack reliable frameworks to distinguish them from actual adaptations-the work presented here provides a plausible mechanism of how such features could arise. To me, this fact alone is interesting, as not many works (as far as I know) have addressed this problem explicitly. Maybe the authors want to emphasize this fact as a novelty of their approach. To be clear, I am not suggesting that the authors should adopt a specific terminology; rather, I believe that explicitly invoking the concept of spandrel would resonate with readers familiar with the foundations of evo-devo and would strengthen the main message of the paper.*

We thank the reviewer for this great suggestion. We have added a reference to Gould & Lewontin's seminal paper in our discussion, placing our findings in the context of spandrels (lines 320-323).

6. *Some additional considerations related to figures*
    a. *Please change colours in the figures to be colour-blind whenever possible*
    b. *The stripes in the striped purple cell shown in Fig. 3A are not seen unless one zooms in on it; would it be possible to represent this differently?*
    c. *In Fig. 5 Aii and Bii, it would be easier for the reader to connect with the statements in the main text if the x-axis is x 1000 or x100 instead of x500*
    d. *Perhaps clarify panel captions of Fig. panels 3C and 3D. Probably I am missing something basic, but I was also wondering how their numbers are connected to the numbers in the panel of Fig. 3F.*
    e. *Why does Fig. 3F have three subpanels? Is it because of different expression levels? Please clarify.*

© 2026. Published by The Company of Biologists under the terms of the Creative Commons Attribution License (https://creativecommons.org/licenses/by/4.0/).

<table>
<tr><td>
A. We thank the reviewer for bringing this up. On revisiting our figures, we noticed some hard-to-distinguish colours for the common red-green colorblindness (deuteranopia). We have improved this by changing the reds closer to magenta, making the figures more accessible.
B. We increased the size of the cartoon cell in Figure 3A and increased the contrast of the colours used to indicate the stripes.
C. We have changed this to read x1000 to improve clarity.
D. We have added the following text to the caption of Fig 3E, page 6, to clear this up: The number in the intersection indicates genes enriched in the boundary compared to both proximal and distal regions.
The numbers within each non-overlapping portion of the circles indicate genes enriched in the boundary relative to only one region (proximal or distal), minus those shared in the intersection.
E. Yes indeed, they represent different order of magnitudes in expression (high, medium, and low, respectively). We have clarified this in the caption of Figure 3F.
</td></tr>
<tr><td>7 . Could the authors clarify the choice of using the Stratonovich approach in the stochastic simulations?</td></tr>
<tr><td>We decided on the Stratonovich interpretation, as it is the interpretation that is most natural when comparing with the deterministic model, where we “turned off” the noise. With the Stratonovich interpretation, we can get a deterministic system by simply dropping the noise terms. Had we chosen the Ito interpretation, this same approach would require changing the dynamics of the deterministic system by including a noise-induced bias in the drift term.</td></tr>
<tr><td>8 . Note equations are referred to in the text as Eq. S (...) whereas they are not supplementary equations</td></tr>
<tr><td>Thanks for pointing this out, we have fixed this in the revised manuscript.</td></tr>
<tr><td>9 . The code is very large (more than 1GB), and I believe much of the space is used by Voronoi tessellations. If the authors have the time and have the scripts generating the Voronoi tessellations, the authors could add them to the repository and ensure that these tessellations are generated during the simulations whenever needed (but I am aware that code organization takes time). I would recommend having the code also in a repository with a DOI (e.g., Zenodo or OSF).</td></tr>
<tr><td>We have reduced the repository size by removing some Voronoi tessellations that are not used in this work, and have created a DOI for the code (line 352).</td></tr>
</table>

Original submission

First decision letter

MS ID#: dev.205745

MS TITLE: Computational Model of Flower Pattern Evolution Predicts Spontaneous Emergence of Boundary Cell Types Across Petal Epidermis

AUTHORS: Steven Oud, Maciej M. Żurowski, Pjotr L. van der Jagt, May T. Yeo, Joseph F. Walker, Edwige Moyroud and Renske M.A. Vroomans

© 2026. Published by The Company of Biologists under the terms of the Creative Commons Attribution License (https://creativecommons.org/licenses/by/4.0/).

Dear Dr Oud,

Thank you for sending your manuscript to Development through Review Commons.

I have now received all the referees reports on the above manuscript, and have reached a decision. The referees' comments are appended below.

The overall evaluation is positive and we would like to publish a revised manuscript in Development.

As you will see, one reviewer commented that this work, as a computational study, is difficult to experimentally verify. I would like you to add a short "limitations of the study" paragraph to the text to address this issue and suggest what type of information/experiments would enable effective testing and refining of the concepts here. This modification would be sufficient in my opinion to address this concern and mover your work to acceptance. You may wish to also consider the points raised by reviewer 3, and to fix the typos they noted.

Reviewer 1: SUMMARY OF THE ADVANCE MADE IN THIS PAPER AND ITS POTENTIAL SIGNIFICANCE TO THE FIELD

This manuscript presents a fascinating modeling exploration of the gene regulatory networks that could evolve to set up a bullseye pre-pattern of pigment in a flower. The authors used a novel UMAP dimensionality reduction similar to single cell RNA-seq to identify different cell types in the models. The authors have added analyses of the breadth of networks that evolve in their models as well as the motifs in these networks, which provide a lot of food for thought.

SUGGESTIONS TO AUTHORS

The authors have thoroughly addressed all of my comments. Congratulations to the authors for their intriguing findings.

Reviewer 2: SUMMARY OF THE ADVANCE MADE IN THIS PAPER AND ITS POTENTIAL SIGNIFICANCE TO THE FIELD

This is a computational study relying on a large number of assumptions and parameter choices, with no obvious way to fully assess the robustness of the paper's conclusions. It would certainly be of interest to researchers working on "artificial life" or adjacent topics.

SUGGESTIONS TO AUTHORS

Please consider whether the peculiar choice of GRN model was definitely the best possible in this context, and to which extent this impacts the results of the paper.

Reviewer 3: The authors have successfully addressed most of my main concerns and the clarity of the manuscript has improved substantially.

- I appreciate the clarification of how cell types are defined, particularly the acknowledgment that this remains an active area of discussion and that clear-cut definitions inevitably involve some degree of arbitrariness. Because this issue is central to the study, operational definitions are necessary and should be tailored to the specific questions being addressed. I think the revised manuscript now acknowledges this point with more transparency.

- Related to the previous comment, the authors might find this article relevant for possible continuations of this work (if they don't know it already): https://www.nature.com/articles/nrg.2016.127. The idea put forward is that novel cell types

© 2026. Published by The Company of Biologists under the terms of the Creative Commons Attribution License (https://creativecommons.org/licenses/by/4.0/).

evolve when ancestral cell types split and become genetically unique through changes in regulatory gene networks, acquiring new identities with specialized functions. I believe the results of this manuscript are consistent with this view.

- I also appreciate the addition of new Methods sections, as well as the streamlining of the existing ones, which improved the readability and clarity of the manuscript.

- Maybe I should clarify the questions I had in interpreting gene profiles I and II. As I understand the authors' definition, the novel cell type in GPI emerges because some genes are preferentially expressed in the boundary, while in GPII, the novel cell type emerges as a combinatorial overlap of different expression regions. I still find this distinction somewhat arbitrary, as the classification depends on how one chooses to interpret the underlying spatial organization. To me, both cases may simply represent different ways in which positional information generates different spatial regions. The fact that gene 0 forms a gradient of expression biases the location where the new cell type can occur. What the rest of the network does therefore is to react/amplify the effect of this gradient to generate sharp boundaries and the subsequent pattern. In any case, I think the addition of the Videos showing the developmental dynamics is very illuminating. From what I can see, I would dare to say that GPI emerges purely from positional information, where the preexisting gradient specifies the location of the novel cell type. In contrast, the expression dynamics of GPII (at least in Video S2) look more like the self-organized establishment of polarity through Turing-like dynamics (the characteristic wavelength of the pattern is on the same scale as the system itself), which appears as a bullseye pattern due to the system's geometry. If that is the case and this is generalizable to all GPII, then I would indeed consider the two mechanisms fundamentally distinct. I mention all this not because I want the authors to further comment on this in the manuscript, but simply because I think it's an issue the authors might want to test in future studies. It could be very interesting, for instance, to take the parameter set used in simulations such as Video S2 and simulate the network spatiotemporal dynamics in larger tissues to determine whether periodic patterns (e.g. spots or stripes) emerge. If that is the case, I believe this could provide an interesting link between the upstream prepatterning mechanisms and the subsequent anthocyanin patterning.

A few typos:

- Line 264: should be "are" and "emphasize" instead of "is" and "emphasises", as it refers to "The recent findings...".
- Line 309: should be "establish" not "establishes".

Overall, I enjoyed reading the revised manuscript, and I believe the authors have addressed my and the other reviewers' concerns, improving the overall quality of the manuscript. Therefore, I think it is suitable for publication in Development.

First revision

<u>Author response to reviewers' comments</u>

Dear Editor,

Thank you for your positive assessment of our manuscript and for the opportunity to revise. We are pleased to address the points raised.

Following your suggestion, we have added a short "Limitations" paragraph to the manuscript discussion. This discusses some current limitations of the model and outlines the types of experiments and empirical data that would allow the findings presented here to be tested and refined.

We have also considered the points raised by Reviewer 3 and corrected all typos they noted.

© 2026. Published by The Company of Biologists under the terms of the Creative Commons Attribution License (https://creativecommons.org/licenses/by/4.0/).

Finally, the abstract has been shortened to comply with the journal's word limit.

We hope the revised manuscript is now suitable for acceptance.

Second decision letter

MS ID#: dev.205745R1

MS TITLE: Computational Model of Flower Pattern Evolution Predicts Spontaneous Emergence of Boundary Cell Types Across Petal Epidermis

AUTHORS: Steven Oud, Maciej M. Żurowski, Pjotr L. van der Jagt, May T. Yeo, Joseph F. Walker, Edwige Moyroud and Renske M.A. Vroomans

Dear Dr Oud,
I am happy to tell you that your manuscript has been accepted for publication in Development, pending our standard publication integrity checks.

© 2026. Published by The Company of Biologists under the terms of the Creative Commons Attribution License (https://creativecommons.org/licenses/by/4.0/).
